# Enhanced stability of a chimeric hepatitis B core antigen virus-like-particle (HBcAg-VLP) by a C-terminal linker-hexahistidine-peptide

**DOI:** 10.1186/s12951-018-0363-0

**Published:** 2018-04-13

**Authors:** Jens Schumacher, Tijana Bacic, René Staritzbichler, Matin Daneschdar, Thorsten Klamp, Philipp Arnold, Sabrina Jägle, Özlem Türeci, Jürgen Markl, Ugur Sahin

**Affiliations:** 1 Biopharmaceutical New Technologies (BioNTech) Protein Therapeutics Corporation, An der Goldgrube 12, 55131 Mainz, Germany; 2 Department of Internal Medicine III, Translational and Experimental Oncology, University Medical Center of Johannes Gutenberg University, Langenbeckstrasse 1, 55131 Mainz, Germany; 3 Institute of Zoology, Johannes Gutenberg University, Johannes-von-Müller-Weg 6, 55128 Mainz, Germany; 4 Ganymed Pharmaceuticals AG, An der Goldgrube 12, 55131 Mainz, Germany; 5 TRON Translational Oncology, University Medical Center of Johannes Gutenberg University, TRON gGmbH, Freiligrathstrasse 12, 55131 Mainz, Germany; 6 Present Address: Anatomical Institute, Otto-Hahn Platz 8, 24118 Kiel, Germany

**Keywords:** Bionanoparticles, Capsid and particle stability, Chimeric VLP, Computational biology, Molecular mechanism, Structural biology, Virus-like-particles (VLP), Vaccine, Hepatitis B virus, Bioengineering

## Abstract

**Background:**

Virus-like-particles (VLPs) are attractive nanoparticulate scaffolds for broad applications in material/biological sciences and medicine. Prior their functionalization, specific adaptations have to be carried out. These adjustments frequently lead to disordered particles, but the particle integrity is an essential factor for the VLP suitability. Therefore, major requirements for particle stabilization exist. The objective of this study was to evaluate novel stabilizing elements for functionalized chimeric hepatitis B virus core antigen virus-like particles (HBcAg-VLP), with beneficial characteristics for vaccine development, imaging or delivery.

**Results:**

The effects of a carboxy-terminal polyhistidine-peptide and an intradimer disulfide-bridge on the stability of preclinically approved chimeric HBcAg-VLPs were assessed. We purified recombinant chimeric HBcAg-VLPs bearing different modified C-termini and compared their physical and chemical particle stability by quantitative protein-biochemical and biophysical techniques. We observed lower chemical resistance of T = 3- compared to T = 4-VLP (triangulation number) capsids and profound impairment of accessibility of hexahistidine-peptides in assembled VLPs. Histidines attached to the C-terminus were associated with superior mechanical and/or chemical particle stability depending on the number of histidine moieties. A molecular modeling approach based on cryo-electron microscopy and biolayer interferometry revealed the underlying structural mechanism for the strengthening of the integrity of VLPs. Interactions triggering capsid stabilization occur on a highly conserved residue on the basis of HBcAg-monomers as well as on hexahistidine-peptides of adjacent monomers. This new stabilization mechanism appears to mimic an evolutionary conserved stabilization concept for hepadnavirus core proteins.

**Conclusions:**

These findings establish the genetically simply transferable C-terminal polyhistidine-peptide as a general stabilizing element for chimeric HBcAg-VLPs to increase their suitability.

**Electronic supplementary material:**

The online version of this article (10.1186/s12951-018-0363-0) contains supplementary material, which is available to authorized users.

## Background

Virus-like-particles (VLPs) are self-assembled, proteinaceous nanoparticles derived from naturally occurring viruses [1]. In last years, VLPs attracted broad scientific interest due to their properties as versatile scaffold in nanotechnology [1–4]. These beneficial characteristics are mainly originated from their particle integrity. Since VLPs are not infectious and do not replicate, they are considered as a safe format in biomedicine [5–8].

One of the best characterized model VLP is based on the wildtype hepatitis B virus core antigen (HBcAg WT) [9, 10]. The HBcAg WT monomer contains an assembly domain (1–149 aa) and a C-terminal domain (CTD) for binding of the nucleic acids. The assembly domain consists of five α-helices and the major immunodominant region (MIR), which is located between α-helix three and four and forms the spikes on the particles. In the assembly domain, cys61 forms an intradimer disulfide-bridge, which is not essential for capsid formation. HBcAg monomers associated into dimers and spontaneously assemble via interdimer contacts into small and large VLP capsids composed of 180 (T = 3-symmetry) or 240 (T = 4-symmetry) subunits [11–13]. It has been hypothesized that T = 3 particles may be more stable as they better tolerate stress-inducing epitope insertions [14]. HBcAg WT lead predominantly (> 90%) to T = 4 capsids [15, 16].

Virus-like-particles per se are robust structures [17–19], and are highly immunogenic as the extremely repetitive and dense presentation of epitopes on the VLP surface efficiently stimulates B cells [5]. When foreign epitopes are introduced into the MIR at the tips of the VLP spikes to obtain so called “chimeric VLP” for immunization, the T = 3/T = 4 capsid ratio can be altered, and the ability to build stable particles can be dramatically decreased [9, 14, 20–22]. As the integrity of VLPs is critical for their immunogenicity [23], strategies to prevent destabilization of chimeric HBcAg-VLPs have been developed [24–26]. The majorities of these alterations implicate disadvantages in vaccine development as they directly affect the foreign epitope sequence and may negatively change the epitope conformation. In addition they are located on a prominent area (e.g. MIR or spike) of the VLP surface, where they tend to display an unwanted, own immunogenicity. Therefore we have focused on novel capsid stabilizing sites/elements located proximal of the VLP to address these problems. An attractive element is a naturally occurring disulfide-bridge (cys61) which is situated inside the HBcAg-structure. Another interesting modification site of the HBcAg is the C-terminus, since it may be located in the particle lumen, has little interaction with the spike (MIR)-inserted epitope and is an essential determinant of VLP geometry and assembly [9, 13, 15, 27–31]. One possible modification is the fusion of a polyhistidine-peptide, as polyhistidine-peptides are known to have only marginal structural impact on the protein and a low to moderate immunogenicity [32, 33]. In addition, it is speculated that the C-terminally added hexahistidine-peptide has an impact on the association behavior of HBcAg and shift HBcAg monomers to non-particulate aggregates [34].

**Fig. 1:**
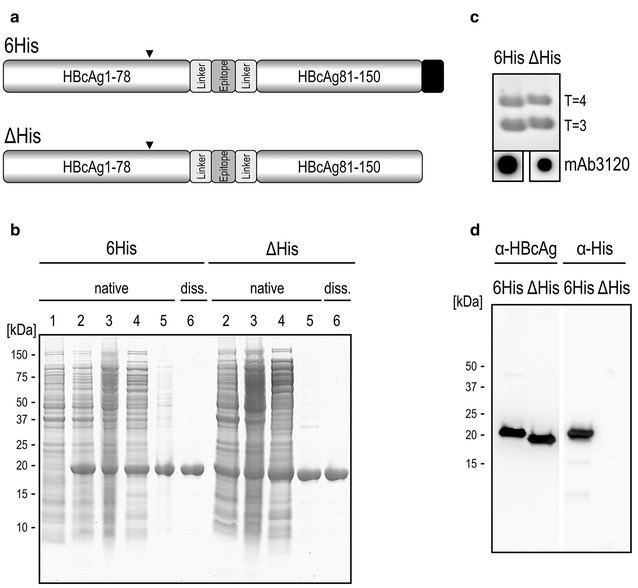
Quality control of chimeric HBcAg-VLPs. **a** 6His is a chimeric HBcAg construct containing a C-terminally located short linker followed by a hexahistidine-peptide (GGSH6, black box), for details see Klamp et al. [38] The chimeric HBcAg construct ΔHis indicates deletion of the linker and polyhistidine-peptide. Arrow head: cysteine residue at position 61. **b** Expression and purification analysis of chimeric VLPs by 12% NuPAGE stained with colloidal Coomassie. Lanes 1 and 2: whole cell lysates from non-induced (1) and induced cells (2), respectively. Lanes 3 and 4: supernatants after removal of cell debris (3) or overnight heat treatment (4), respectively. Lane 5: resuspended protein sediment after AMS-precipitation. Lane 6: purified and reassembled chimeric VLPs. **c** PageBlue stained native agarose gel analysis of HBcAg-VLPs (lane 6 from **b**). The triangulation number of T = 4-VLPs and T = 3-VLPs are designated. The lower row panel represents a dot blot analysis of identical spotted protein amounts of both VLPs, using mAb3120, specifically detecting assembled particles. **d** Polypeptides were identified by immunoblotting of chimeric VLPs (compare lane 6 in **b**) using anti-HBcAg (mAb 2–10aa, α-HBcAg) or anti-hexahistidine-peptide (α-His)

Aim of this study was to investigate the stabilizing effect of a C-terminal, histidine-peptide extension, as well as a conserved intradimer disulfide-bridge on the stability of chemically and physically stressed T = 3 and T = 4 chimeric HBcAg-VLPs. Protein-biochemical and -biophysical methods were employed to allow comparison and semi-quantification of the stability. To investigate this, we used a preclinically evaluated chimeric HBcAg-VLP which is decorated by the extracellular loop of splice variant 2 of claudin 18 (CLDN18.2). Proteins of the claudin family are essential transmembrane components of tight junctions. CLDN18.2 is a highly selective tumor-associated antigen and is significantly expressed in different solid cancer entities (carcinomas of the stomach, pancreas, esophagus and NSCLC) and in normal tissues strictly restricted to gastric mucosa [35]. Therefore, CLDN18.2 is an attractive target for antibody-based therapies, which are currently investigated in a phase III study in solid cancer [36, 37]. Furthermore, CLDN18.2 is also a target for active immunization therapy with VLPs. Such CLDN18.2-bearing VLPs induce immune responses against CLDN18.2 expressing cancer cells and are capable to significantly reduce the formation of pulmonary tumors in a prophylactic vaccination mouse model [38]. The VLP presented in this study (Fig. 1a; 6His) displays a surface epitope of claudin 18.2, and possesses a C-terminal 9mer-peptide consisting of a mini-linker (GGS) and a hexahistidine-peptide. When compared to an identical chimeric VLP without C-terminal modification (Fig. 1a; ΔHis) it assembles in a similar T = 3/T = 4-symmetry ratio (Fig. 1c; Table 1) and was therefore chosen as model to analyze the T = 3/T = 4-integrity promoting influence of the hexahistidine-peptide. Up to now, the stability of chimeric HBcAg-VLPs has not been thoroughly studied as to the author’s knowledge. To achieve a comprehensive data set on the particle integrity of chimeric HBcAg-VLPs, we chose a multivariate, complementary analysis of stressed VLP samples. Selection of the analysis methods was based on sample throughput; techniques should provide independent results from each other and a broad size range (VLP [nm] to aggregates [µm]) should be covered. We further extended the analyses to a second, proprietary chimeric HBcAg-VLP to demonstrate that the hexahistidine-peptide mediates stability in general.

**Table 1:** VLP properties

	NAGE^a^		TEM^b^		DLS^c^	
	6His	ΔHis	6His	ΔHis	6His	ΔHis
T = 4 ratio [%]	48.1 ± 2.1	48.2 ± 3.0	43	43	39.9 ± 5.8	41.5 ± 6.4
T = 4 diameter [nm]			33.8 ± 0.1	30.9 ± 0.3		
T = 3 diameter [nm]			29.1 ± 0.1	26.8 ± 0.3		

^a^ From densitometry measurements

^b^ For estimation of the ratio of the individual particle symmetries (T = 4; T = 3), a collective of 1700 particles were counted

^c^ Hydrodynamic diameter; no discrimination between T = 3 and T = 4 was possible

In this paper we demonstrate that chimeric HBcAg-VLPs are stabilized against diverse stress conditions by addition of a C-terminal polyhistidine-peptide and/or a disulfide-bridge. These findings may be of high importance for future HBcAg-based nanostructural design.

## Results

### Expression, purification and structural analysis of chimeric HBcAg-VLPs

The objective of this work was to analyze the impact of a C-terminally located 9mer-peptide, consisting of a short linker and a hexahistidine-peptide (GGSH6), on the stability of a chimeric HBcAg-VLP based vaccine candidate for cancer immunotherapy [38]. For this purpose, two chimeric VLP variants were generated, one without (ΔHis) and one with a C-terminal hexahistidine-peptide (6His) (Fig. 1a). Both constructs were expressed in *E. coli* and purified both under native and under capsid dissociating conditions (Fig. 1b). Preparations purified under native conditions contained host cell protein contaminations, whereas the dissociating purification process yielded VLPs of near homogeneity (Fig. 1b). Those preparations exhibited a purity of > 90% as measured by densitometric SDS-PAGE analysis and negligible amounts of residual host cell DNA or endotoxin contaminations (Additional file 1: Table S1) and therefore fulfill criteria for a phase I clinical trial in patients. Purified VLPs migrated as distinct protein bands with the expected differences in migration behavior in SDS-PAGE and NAGE (Fig. 1b, c). The identity of purified chimeric HBcAg-VLPs was confirmed by immunoblot analysis with two highly specific monoclonal antibodies, either recognizing the N-terminus of both constructs or the C-terminus of the 6His construct (Fig. 1d). The immunoblot result was supported by mass spectrometric analysis, yielding peptide fragments that cover most of the protein sequences, including the C-termini of both constructs (Additional file 1: Table S2).

**Fig. 2:**
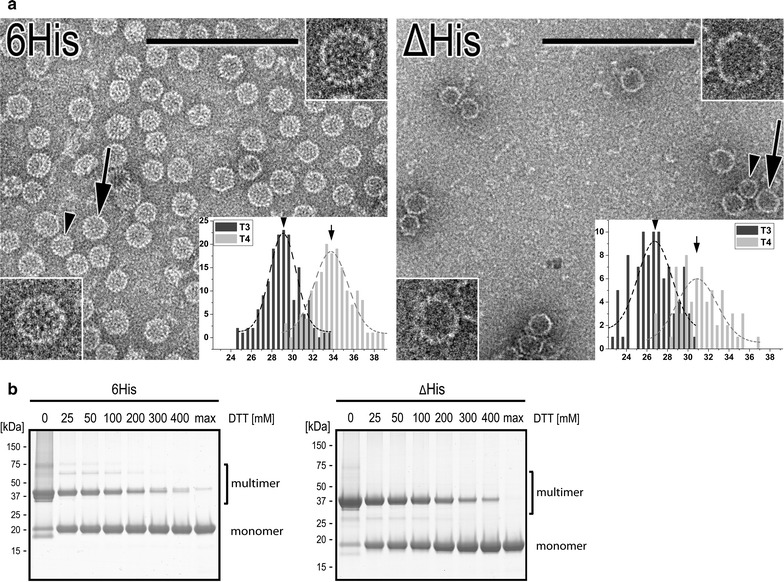
Initial characterization of purified chimeric VLPs by TEM (transmission electron microscopy) and non-reducing PAGE. **a** TEM of negatively stained VLP preparations (from lane 6 in Fig. 1b). T = 4-VLPs are marked with arrows (for higher magnification see inset in the upper right corner); T = 3-VLPs are labeled with arrow heads (for higher magnification see insets in the lower left corner). Scale bars: 200 nm. Histogram insets show diameter distributions for T = 4- (light grey) and T = 3-VLPs (dark grey). Abscissa: diameter in [nm]; ordinate: VLP counts. Only entirely intact particles were counted, total number was 479. The VLP mean diameters were fitted by Gaussian curves and the maxima are indicated in Table 1. **b** Colloidal Coomassie stained 12% NuPAGE analysis of VLPs (from lane 6 in Fig. 1b) treated with increasing DTT-concentrations. Samples were incubated with or without DTT 15 min prior non-reducing buffer supplemented and heated to 60 °C, max: sample in reducing buffer after boiling. Mono- and multimeric forms of chimeric VLPs are indicated

The particulate structure of the purified VLPs was verified independently by approved techniques, namely DLS, NAGE, dot blot analysis and negative staining TEM (transmission electron microscopy) analysis (Table 1) [16, 39–41]. Ultrastructural analysis of VLPs by TEM indicated a homogenous diameter distribution of the 6His-VLPs, centered at 33.8 and 29.1 nm for T = 4- and T = 3-particles and consisted of uniform and regular particles. Whereas ΔHis-VLPs exhibited a slightly more heterogeneous diameter distribution, centered at 30.9 nm for T = 4- and 26.8 nm for T = 3-particles, some particles were misshaped and partially disrupted (Fig. 2a, insets and Table 1). A similar result was obtained by dot blot analysis detecting capsid-conformational epitopes using the mAb3120 (Fig. 1c). The spotting of identical amounts of VLPs resulted in about half of the signal intensity for the ΔHis VLPs than observed for the 6His-VLPs. However, these differences could not be detected when analyzing the VLPs in solution by DLS or NAGE (Fig. 1c). Unstressed 6His and ΔHis VLPs showed similar T = 3:T = 4 ratios of nearly 50:50 in NAGE, which was confirmed by TEM (Fig. 1c and Table 1).

**Fig. 3:**
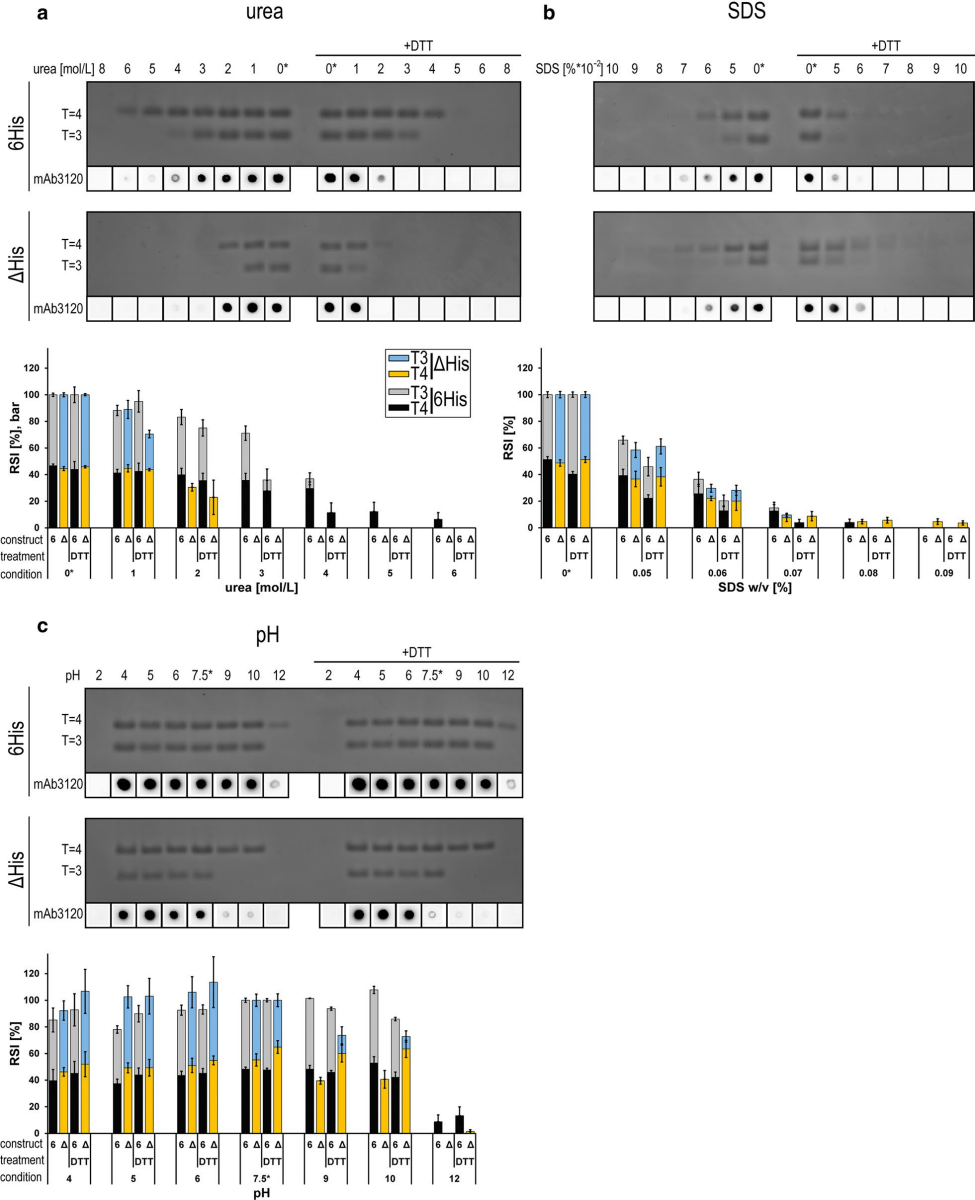
Effects of chemical stress on VLP stability. **a** 6His-VLPs (upper row) and ΔHis-VLPs (lower row) were stressed by chaotropic agent (urea) in the absence or presence (+DTT) of 100 mM DTT and subsequently analyzed on PageBlue-stained 2.6% native agarose gels. Corresponding samples were analyzed by dot blots using mAb3120 antibody specific for assembled VLPs, which is shown below the agarose gel pictures. Bar diagrams show the normalized densitometric analysis of stained protein bands, separated by NAGE. 6His-VLPs (6) are indicated in black (T = 4) and grey (T = 3), ΔHis-VLPs (Δ) in orange (T = 4) and blue (T = 3) bars. Four independent purifications of 6His-VLPs and three independent purifications of ΔHis-VLPs were analyzed and 4–10 stained NAGEs per test were quantified. Untreated reference samples used for normalization are marked with *. Average values are shown and standard deviations are indicated by error bars. Only experimental conditions which resulted in RSI (relative signal intensity) values above zero are specified. **b** 6His-VLPs (upper row) and ΔHis-VLPs (lower row) were stressed by detergent (SDS) and analyzed as in **a**. **c** 6His-VLPs (upper row) and ΔHis-VLPs (lower row) were stressed by different pH and analyzed as in **a**

**Fig. 4:**
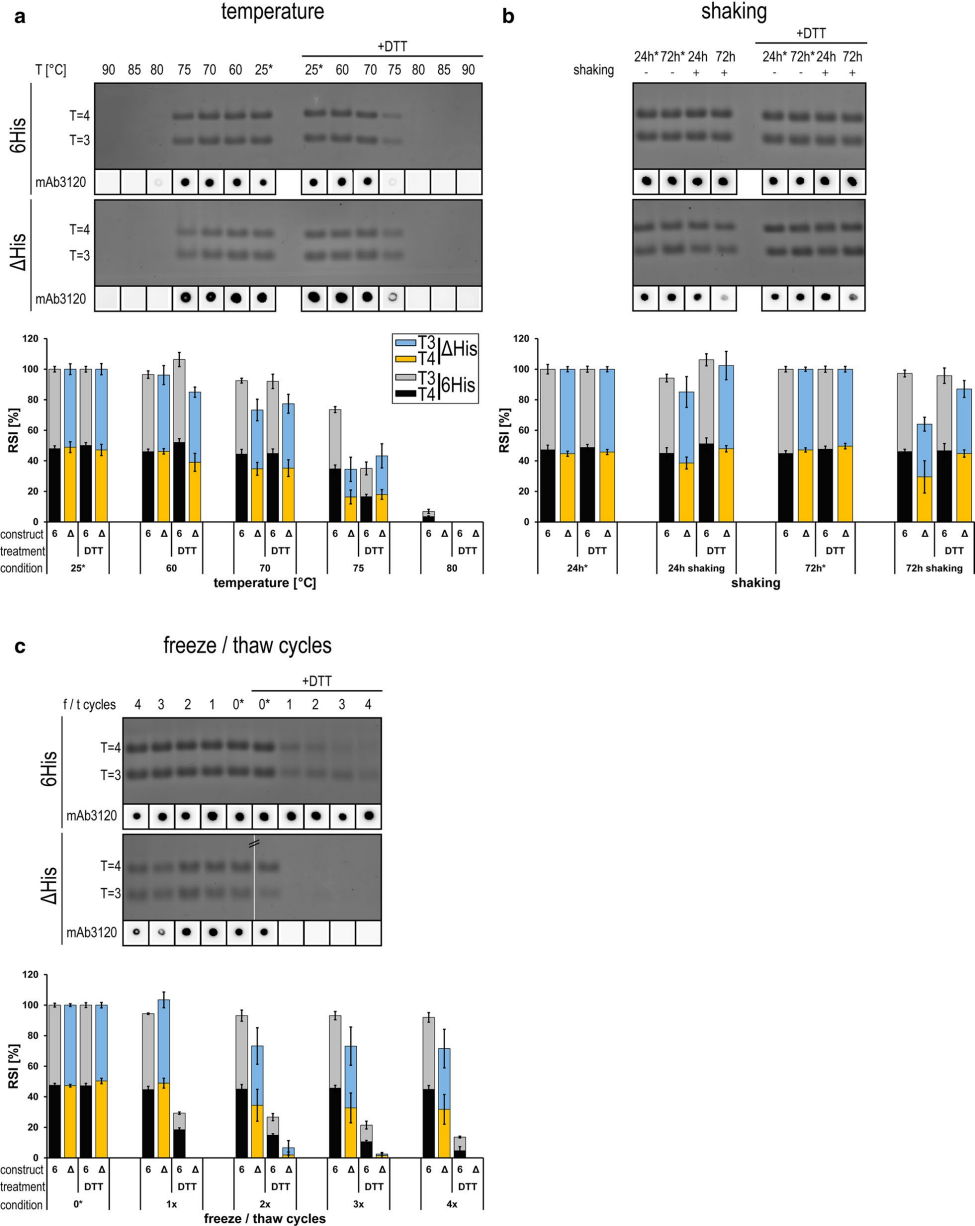
Effects of physical stress on VLPs stability. Following stability tests using **a** temperature, **b** shaking or **c** freeze/thaw cycles NAGE and dot blot were performed (as described in Fig. 3)

To evaluate the influence of the disulfide-dependent HBcAg-multimerization on the stability of chimeric VLPs, we analyzed their occurrence, localization and reducibility in the 6His and ΔHis constructs. Disulfide-dependent chimeric HBcAg-multimers could be separated and detected by non-reducing SDS-PAGE conditions (Fig. 2b). We identified cys61 (Fig. 1a, arrow heads) as the main interaction in disulfide-dependent chimeric HBcAg-multimerization (see “Discussion”). When the reducing agent DTT was added to the chimeric VLPs, a clear decrease of the multimeric protein bands and a simultaneous increase of the monomeric protein bands could be detected by PAGE (Fig. 2b). Subsequent stability tests of chimeric VLPs analyzing the DTT-influence (Figs. 3, 4) were performed at 100 mM DTT. This concentration lead to a significant reduction of the involved disulfide bonds (Fig. 2b and Additional file 1: Figure S1).

In summary, these results indicate that both constructs form highly pure particles which can be further analyzed for their capsid stability.

### Chemical stability of chimeric HBcAg-VLPs with and without hexahistidine-peptide

ΔHis- and 6His-VLPs samples were subjected to various chemical stress conditions and were analyzed for their capsid integrity by three independent approaches, NAGE, DLS, and dot blot. Dot blot data relied mainly on the detection of the spotted VLPs by the mAb3120. This antibody detects a discontinuous, conformational epitope, located on the basis of two monomers assembled in a dimeric trimer VLP-structure [42, 43]. Reduction or loss of dot blot signal therefore indicated any kind of epitope modification, either because the antibody could not bind or because the binding of the HBcAg-sample onto the membrane was affected. On the basis of DLS the hydrodynamic diameter of macromolecules in solution could be deduced. The light scatter intensity was proportional to the average molecular weight of all solutes, and was dominated by the largest and most prominent species. DLS was highly sensitive for large aggregates. HBcAg-particles could be visualized by NAGE [44, 45]. In NAGE, capsids are defined as sharp, stained protein bands, particle aggregation or dissociation is defined as fuzzy banding with decreased intensity or complete loss of signals [44, 46]. Unlike the other methods, such as DLS or dot blot analysis, analytical NAGE was the only technique used for VLP-analysis in this study which reliably discriminated between T = 3- and T = 4-symmetries of HBcAg-VLPs (Fig. 1c) [47]. Therefore we established a semi-quantitative NAGE densitometry which enables both, the quantitative estimation and the qualitative differentiation between chimeric T = 3- and T = 4-capsids.

When the chimeric VLPs were treated with urea, a chaotropic, nonionic protein denaturant, the particle stability was affected in a urea-concentration dependent manner. 6His-VLPs were significantly more resistant than ΔHis-VLPs. The stability of both icosahedral particle symmetries was diminished in the presence of DTT. Furthermore, the capsid stability of T = 3 particles of both constructs dissociate at lower urea concentrations than T = 4 particles. Similar results were obtained by dot blot analysis using mAb3120 specific for assembled capsids (Fig. 3a). Moreover, hydrodynamic diameter measurements by DLS showed an almost constant mean diameter of 40 nm from 6His in the presence of up to 4 M urea, corresponding to capsids still in 4 M urea solution. The ΔHis construct on the other hand exhibited sufficient scatter values only up to 1–2 M urea concentration indicating lower particle stability in solution (Additional file 1: Figure S2A).

As a second chemical stress test, the influence of an ionic detergent (SDS) at concentrations between 0.01 and 0.1% on the stability of 6His and ΔHis capsids was analyzed (Fig. 3b). Until a SDS concentration of 0.04% only a minor influence on the particle integrity was detectable, at SDS concentrations above 0.05% the capsid integrity started to be affected. Both 6His and ΔHis capsids showed a comparable stability. As observed before, particles of both constructs with T = 3 symmetry exhibited a weaker stability than T = 4 capsids. The presence of DTT had a minor destabilizing effect on particles. Dot blot results were congruent with the NAGE results. DLS analysis showed an almost constant radius until a concentration of 0.06% (Additional file 1: Figure S2A).

We next examined the VLP stability of 6His and ΔHis over a broad pH range. At acidic ranges, both constructs were similarly stable (Fig. 3c) and particle integrity was lost only at a pH of two (indicated by faster migrated smear in NAGE, data not shown). In contrast, at moderate alkaline pH, the 6His-VLPs were more stable than the ΔHis-VLPs, again with an overall higher stability of T = 4 capsids, which was more pronounced for the ΔHis-VLPs. Similar observations could be done by dot blot analysis, although binding of the mAb3120 seemed to be generally stronger influenced by the experimental conditions than NAGE. T = 4 particles appeared to be less detected by the mAb (Fig. 3c, at alkaline pH) compared to the urea or SDS treated samples (Fig. 3a, b). Although the ΔHis DTT reference sample produced lower signal intensity, the presence of DTT had only a low effect on the VLP integrity at alkaline pH values (Fig. 3c, bar diagram). DLS-measurements indicated an increase of the mean diameter at acidic pH values that correspond to isoelectric point precipitates of the chimeric VLPs (Additional file 1: Figure S2A).

Overall, all analyzed chemical stress parameters have an impact on the particle stability of chimeric VLPs and DTT treated samples showing a lower stability in urea- and SDS-tests. In general T = 3 particles show a minor stability compared to T = 4 particles (Additional file 1: Figure S3A). In summary, the 6His construct proves a higher chemical robustness compared to the ΔHis construct, and stability by the addition of the C-terminal 9mer-residues increases sequential: 6His T = 4 > 6His T = 3, ΔHis T = 4 > ΔHis T = 3. These observations let us conclude a particle stabilizing influence of the linker-hexahistidine-peptide.

### Physical stability of chimeric HBcAg-VLPs with or without hexahistidine-peptide

In addition to chemical stress tests, we also examined the influence of diverse physical stress parameters on the stability of 6His and ΔHis particles. 6His and ΔHis capsids could not be detected by NAGE and dot blot when treated with a temperature higher than 80 °C, ΔHis particles exhibited a lower thermal stability than 6His capsids (Fig. 4a, see 70–80 °C bar diagram). Interestingly, no difference between T = 4 and T = 3 capsids was apparent in NAGE analysis and the presence of DTT only slightly diminished the stability of the capsids. Heat treatment at temperatures of 70 °C and beyond leads to thermal aggregation of the chimeric VLPs. That can already be recognized by the appearance of white precipitates in stressed 6His construct samples, interestingly this was not seen for the ΔHis construct. Heat induced particle aggregation could also be clearly observed by DLS analysis showing an increase of the particle mean diameter beginning at 70 °C, that was more pronounced in the presence of DTT (Additional file 1: Figure S2B).

According to NAGE and dot blot analysis, shaking for up to 72 h at 25 °C did not influence the stability of 6His-VLPs, whereas a slight negative effect was detectable for ΔHis particles after 72 h of shaking that again equally affected T = 3 and T = 4 capsids (Fig. 4b, bar diagram). This effect could be observed distinctly for the ΔHis-VLPs by dot blot analysis, leading to faint signals at 72 h of shaking (Fig. 4b). The presence of DTT had a positive effect on the capsid integrity (Fig. 4b, bar diagram). DLS measurements showed an increase of the mean diameter after 72 h w/o shaking (Additional file 1: Figure S2B) compared to fresh samples (Table 1). This could arise from self-aggregation of VLPs over time in Tris-buffer, which can be avoided by addition of DTT.

As a third physical stress parameter, the influence of varying numbers of repeated freeze/thaw cycles on VLP stability was analyzed (Fig. 4c). Concluding from NAGE analysis, 6His capsid particles were highly resistant to multiple freeze/thaw cycles in the absence of reducing agents. Minimal influence was detectable by NAGE and only a slightly less intensive dot blot signal after four freeze/thaw cycles could be observed. The T = 3:T = 4 ratios of both constructs remained again constant for all conditions investigated (Additional file 1: Figure S3B). ΔHis capsids were more prone to freeze/thaw stress than 6His-VLPs which was consistent with their overall weaker stability already observed in most of the previous stress tests. This effect was in particular visible in the presence of DTT, even a single freeze/thaw cycle led to the complete disappearance of NAGE or dot blot signals of the ΔHis construct. DLS measurements of samples in the presence of DTT showed a strong increase of the mean diameter which was much earlier detectable at the ΔHis samples (Additional file 1: Figure S2B).

In contrast to chemical stress, in physical stress-tests no differences in the T = 3:T = 4 ratio could be detected (Additional file 1: Figure S3B) and again a sequential stability was observed: 6His > ΔHis. Overall these experiments confirm the observation that DTT-sensitive disulfide bridges lead to a general stabilization of the VLP integrity, furthermore the linker-hexahistidine-peptide leads to stabilization even under physical stress.

### Impact of the polyhistidine-peptide-length on the stability of chimeric HBcAg-VLPs and stabilizing effect of the peptide on a second chimeric VLP

**Fig. 5:**
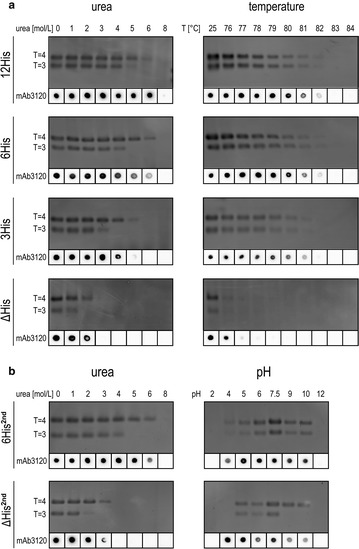
Effects of physical and chemical stress on the stability of chimeric VLPs with different polyhistidine-peptide lengths and on an alternative chimeric VLP. **a** Chimeric VLPs containing 0; 3; 6; 12 histidine residues (ΔHis, 3His, 6His, 12His) at the C-terminus were chemically (urea) or physically stressed (temperature). Effects on the capsid stability were analyzed by NAGE and dot blot (for details see Fig. 3). **b** Chimeric VLPs (6His^2nd^/ΔHis^2nd^) with a different epitope sequence and MIR-insertion site were chemically and physically stressed as described before, only stress-tests resulting in significant differences of the VLP stability are shown (urea and pH). All assays were performed in triplicates

The previous experiments demonstrated that the integrity of the chimeric particles is not only stabilized by disulfide bridges but also by the presence of a C-terminally located linker-hexahistidine-peptide. In order to analyze the linker-hexahistidine-peptide mediated stabilizing effect in dependency on the histidine-peptide length, we generated additional chimeric HBcAg-VLPs, differing in the number of C-terminal histidines (3 and 12 histidines; depicted as 3His and 12His). As delineated above the most significant effects on the chemical and physical particle stability of 6His and ΔHis were observed in urea and temperature stress tests (Figs. 3, 4). Therefore, these two tests were repeated using the ΔHis, 3His, 6His and 12His constructs purified under native conditions. Again, samples were stressed with varying urea concentrations (Fig. 5a, left panel). For both particle symmetries a histidine-peptide length dependent, sequential stability was observed by NAGE and dot blot analysis, with 12His-VLPs being the most stable and ΔHis-VLPs being the least stable particles. Interestingly, already three C-terminal histidines were sufficient to obvious stabilize the particle integrity. Between six and twelve C-terminal histidines only a marginal increase of the capsid stability was observed in NAGE (compare 5 M urea samples), which was better visible in dot blot results (5 M vs 6 M urea samples). Under chemical stress (Fig. 3), NAGE showed a generally higher stability for T = 4 compared to T = 3 particles independent of the polyhistidine-peptide length, which was noticed before.

The temperature stress test were closely examined, samples were exposed to different temperatures above 76 °C in steps of 1 °C. Again, a sequential stability of the four VLP constructs could be observed: 12His, 6His > 3His > ΔHis. No significant difference between T = 4 and T = 3 stability was observed (Fig. 5a, right panel); this was in agreement to the previous thermal/physical tests (Fig. 4a).

The results presented in this paragraph demonstrate that the stabilizing influence of the linker-polyhistidine-peptide is dependent on the histidine moieties. The longer the linker-polyhistidine-peptide is, the more stable the chimeric VLPs are.

To investigate whether the C-terminal 9mer-peptide (GGSH6) exerts its stabilizing influence also on other HBcAg-VLPs, and therefore show a transferability of the peptide-mediated stabilizing effect, all stress-tests were repeated with a second chimeric VLP pair (6His^2nd^/ΔHis^2nd^). The additional 6His^2nd^/ΔHis^2nd^ constructs deviate from 6His/ΔHis but varied in the epitope sequence and furthermore in the epitope insertion site. Here, a novel epitope was inserted, resulting in particles with different behavior (e.g. higher electro mobility) in contrast to the previous used chimeric VLPs. In four out of six stress tests a similar stability of both constructs were observed (data not shown). Remarkably, similar results as for the original 6His and ΔHis constructs were obtained in urea and pH stress test (Fig. 5b). Again, the particles with C-terminal located linker-hexahistidine-peptide were significantly more stable than the particles without the peptide. As found for the initially investigated particles the T = 4 particles are also more robust than the T = 3 particles.

Therefore, it can be concluded that the linker-hexahistidine-peptide-mediated stabilizing effect is of general nature, and can be transferred to other chimeric HBcAg-VLPs.

### Protein simulations of the C-terminus

**Fig. 6:**
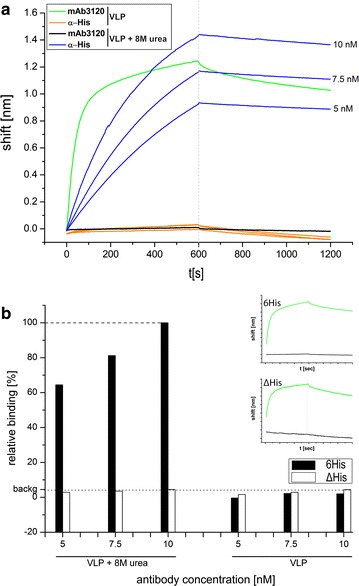
Accessibility of the C-terminally located hexahistidine-peptide on chimeric HBcAg-VLPs. **a** Typical plot of biolayer interferometry measurement (6His construct). All measurements were performed using equal VLP-loadings of 1.6 nm ± 0.1 on APS-sensors. Green and black curves indicate the detection of intact (green) or disassembled (black) VLPs using the mAb3120 specific for assembled particles. Blue and orange curves show detection of the hexahistidine-peptide on intact (orange) or disassembled (blue) VLP with an anti-hexahistidine-peptide antibody (α-His) in a concentration dependent manner. **b** Bindings of the α-His antibody to chimeric VLPs purified under native conditions. Bars indicate response rates at 595 s. The response rate of 6His + 8 M urea at antibody concentration of 10 nM was set to 100% binding (dashed line) and the rate of ΔHis + 8 M urea at the same antibody concentration was set as background (backg, dotted line). Insets display mAb3120 control plots using VLPs with or without 8 M urea (same color as in **a**)

We hypothesized that the particle stabilizing effect of the linker-hexahistidine-peptide in chemical and physical stress tests may rely on intra- and/or intersubunit interactions of the hexahistidine-peptide with residues of the HBcAg-backbone molecule. To analyze this hypothesis, protein simulation studies of the C-terminal linker-hexahistidine-peptide in context of the capsid were performed based on experimental data, to deduce possible stabilizing interactions. To decrease the simulated area, we first analyzed the localization of the C-terminal hexahistidine-peptide in- or outside of the particle lumen. For this purpose, accessibility studies of the hexahistidine-peptide on immobilized intact (VLP) or disassembled (VLP + 8 M urea) chimeric particles were performed, using two specific antibodies in a biolayer interferometry (FortéBIO Octet Red) experiment (Fig. 6). As expected, the signals for disassembled or intact VLPs without the hexahistidine-peptide (ΔHis) were very low when the anti-His6-peptide antibody for detection was used (Fig. 6b, white bars), indicating a minor nonspecific binding of the antibody and the signal was therefore set as background. Not surprisingly, a different picture arose for 6His particles. Denaturation of the particles would lead to an increased accessibility of the hexahistidine-peptide and, therefore, concentration dependent binding of the anti-His6-peptide antibody was expected. Disassembled 6His particles indeed lead to a strong concentration dependent binding of the anti-His6-peptide antibody to the now accessible hexahistidine-peptide. Only very low signal intensities, below the background level of the anti-His6-peptide antibody was measurable for intact 6His-VLPs (Fig. 6), demonstrating inaccessibility of the hexahistidine-peptide in assembled 6His HBcAg-VLPs. These combined observations of our biolayer interferometry data and cryo-electron microscopy results (see below) indicate a luminal orientation of the hexahistidine-peptide in chimeric VLPs. This is in agreement with previous published data on HBcAg-VLPs.

**Fig. 7:**
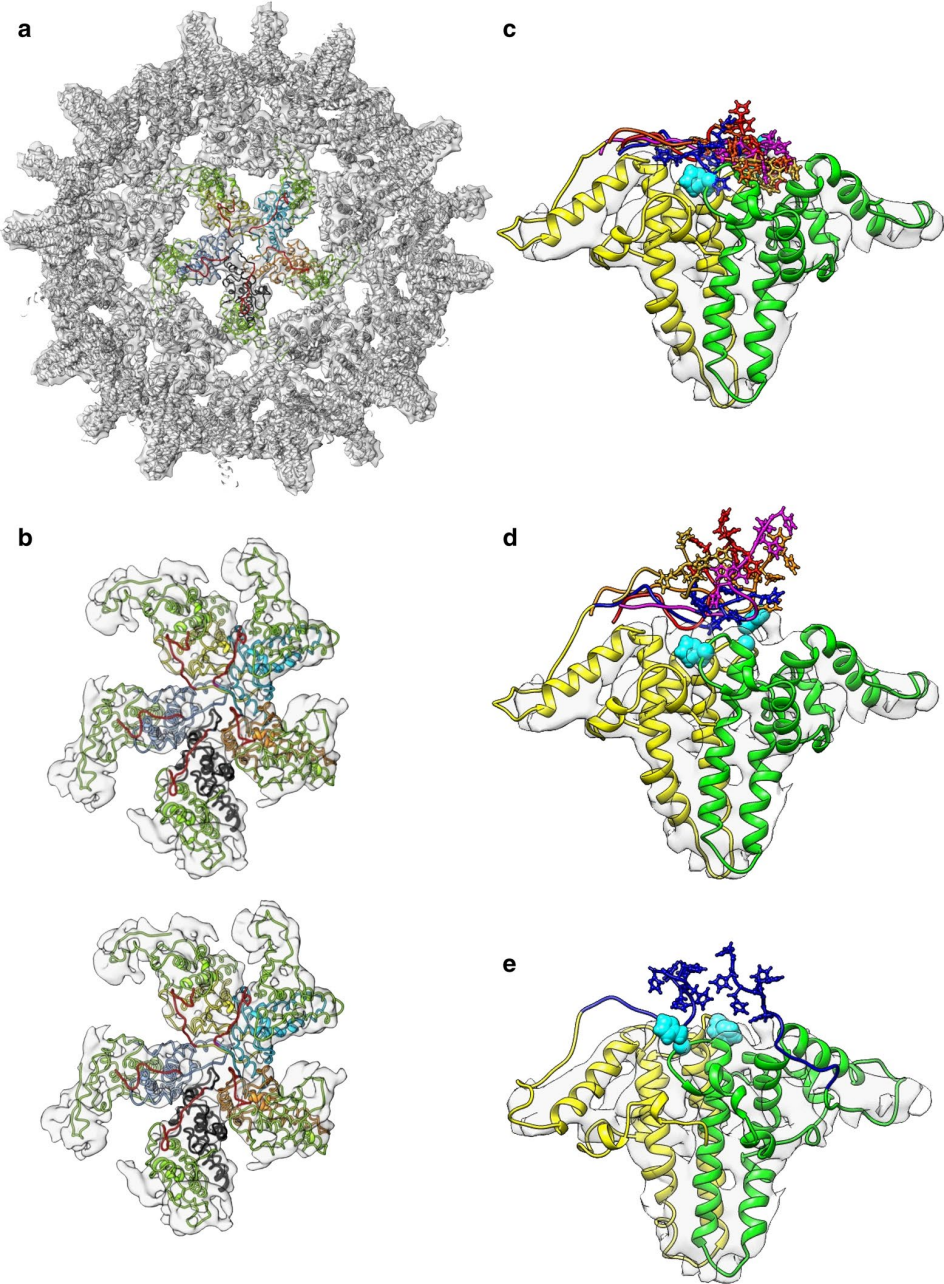
Simulation of the C-terminal linker-hexahistidine-peptide of chimeric 6His-VLPs. **a** 3D reconstruction of the chimeric 6His-VLP where the C-terminus is simulated by MD with density map present. The view is inside of the VLP lumen, half capsid density map (gray) and fitted crystal structure of a HBcAg dimer (PDB ID: 1QGT, gray ribbons). Penta-dimer structure in colored ribbons, green distal monomers and rainbow colors proximal monomers from which the C-terminal ends (LPETTVVRGGSHHHHHH, red) are simulated. **b** Stereo pair of the penta-dimer structure. For improved visualization of the C-termini, a slightly tilted and magnified view is shown. **c** Orientation of the C-terminus of the 6His-construct with the density map present during the MD simulation. The crystal structure of the HBcAg dimer (yellow and green ribbons) with his47 (cyan) in space fill is shown. Five penta-dimer MD calculated positions of the C-terminus are shown (yellow, green, blue, red and magenta). The hexahistidine-peptide is represented by ball and stick model. Note that the hexahistidine-peptide is in close proximity to the dimer basis and to his47. **d** Orientation of the C-terminus of the 6His-construct by MD simulation after removal of the density map factor, colors are as in **c**. Note fewer interactions of the histidines from the hexahistidine-peptide with the histidines found in the native HBcAg sequence (e.g. his47). **e** MD simulation of single dimer with two C-terminal hexahistidine-peptides present on each monomer

In order to obtain possible 3D structures of the C-terminal hexahistidine-peptide and hereby assess potential stabilizing interactions at sub macromolecular level, we performed MC (Monte Carlo) and MD (Molecular Dynamics) simulations in combination with a density map derived from 6His-HBcAg cryo-electron microscopy images. From 1800 single particles extracted from cryo-electron microscopical images of VLP, a 3D reconstruction with a final resolution of 7 Å was produced. The published crystal structure of the HBcAg dimer fitted well into the obtained density map (Fig. 7a). To get reasonable starting structures for the subsequent simulations, we started with MC calculations which explored the large conformational space of an HBcAg-penta-dimer structure with internal situated C-termini (Fig. 7a, colored ribbons). MC generated models were the initial structure for the MD simulations; as expected MDFF (Molecular Dynamics Flexible Fitting) attracted the carboxy-terminal parts of the 6His-VLP into the density map (Fig. 7a–c, Additional file 2: Movie S1). In order not to overrate the impact of the density map, we chose a small density potential. Unrealistic or inconsistent conformations created by MDFF did not remain stable during the free MD simulations without density map, and thus could be excluded. The remaining conformations denoted an interaction of the hexahistidine-peptide with the dimer basis facing the lumen of the particle (Fig. 7d). Surprisingly, no cross interaction with proximate dimers could be observed (Fig. 7b). We found an approximation of the first histidine from the hexahistidine-peptide with the dimer basis, mainly to his47 (Fig. 7c, d, cyan space fill). His47 was founded to be conserved, and was located in a loop, between the α2 and α3 helix and might be flexible enough to interact with the hexahistidine-peptide [13]. Although the non-conserved residues his51 and his52, located in the border of the α3 helix, were also in close proximity, they may not be the primary interaction partners. Subsequent histidine residues of the hexahistidine-peptide oriented to the C-terminus were more flexible and frequently left the close vicinity of the dimer surface (Fig. 7d) but could interact with histidines of the partner HBcAg-monomer, especially with his47. Free MD simulation of the C-terminus without hexahistidines (ΔHis) was performed for comparison. Here, the C-terminus was commonly expelled from the density map (data not shown). The fact that the first histidine of the peptide was able to interact with the HBcAg-dimer basis is in agreement with experiments where already three histidines (3His) exerted a stabilizing effect on the capsid integrity (Fig. 5a).

From free MD simulations (Fig. 7d), it became obvious that also the C-terminal histidines of two 9mer-peptides may interact within one dimer at the interface region. To further investigate this, we repeated the simulations using a single dimer with C-terminal linker-hexahistidine-peptides present in each monomer (Fig. 7e). 40% of all MD simulations in combination with density map indicated approximation of distal histidines of the two peptides with each other. The presented results lead to the assumption that additional C-terminal histidines (e.g. 6 and 12His) could interact with each other and further stabilize the integrity of the VLP.

In summary, we developed a molecular simulated model for the hexahistidine-peptide-dependent particle stabilizing effect based on cryo-electron microscopy data, which suggested stabilization via two stages. An interaction of proximal peptide-histidines with the HBcAg luminal side mainly via his47 may occur, additionally adjacent hexahistidine-peptides within one dimer may interact.

## Discussion

Employing protein-biochemical and -biophysical methods, we have demonstrated that a C-terminal modification consisting of a short linker peptide (GGS) and a polyhistidine-peptide substantially increases the proportion of assembled chimeric HBcAg-VLPs when subjected to various chemical or physical stress conditions. The stabilizing effect was proportional to the length of the polyhistidine-peptide (increasing stability: ΔHis < 3His < 6His, 12His). Interestingly, T = 3 particles demonstrated a minor chemical resistance compared to T = 4 particles. By utilizing MD simulations which were deduced from cryo-electron microscopy data and supported by biolayer interferometry, we were able to develop a theoretical model where the hexahistidine-peptide interacts on the basis of the HBcAg-mono/dimer and exert stabilizing effects. In addition, a naturally occurring and experimentally confirmed disulfide-bond (cys61) was identified as an additional, independent stabilizing element for chimeric HBcAg-VLPs.

**Fig. 8:**
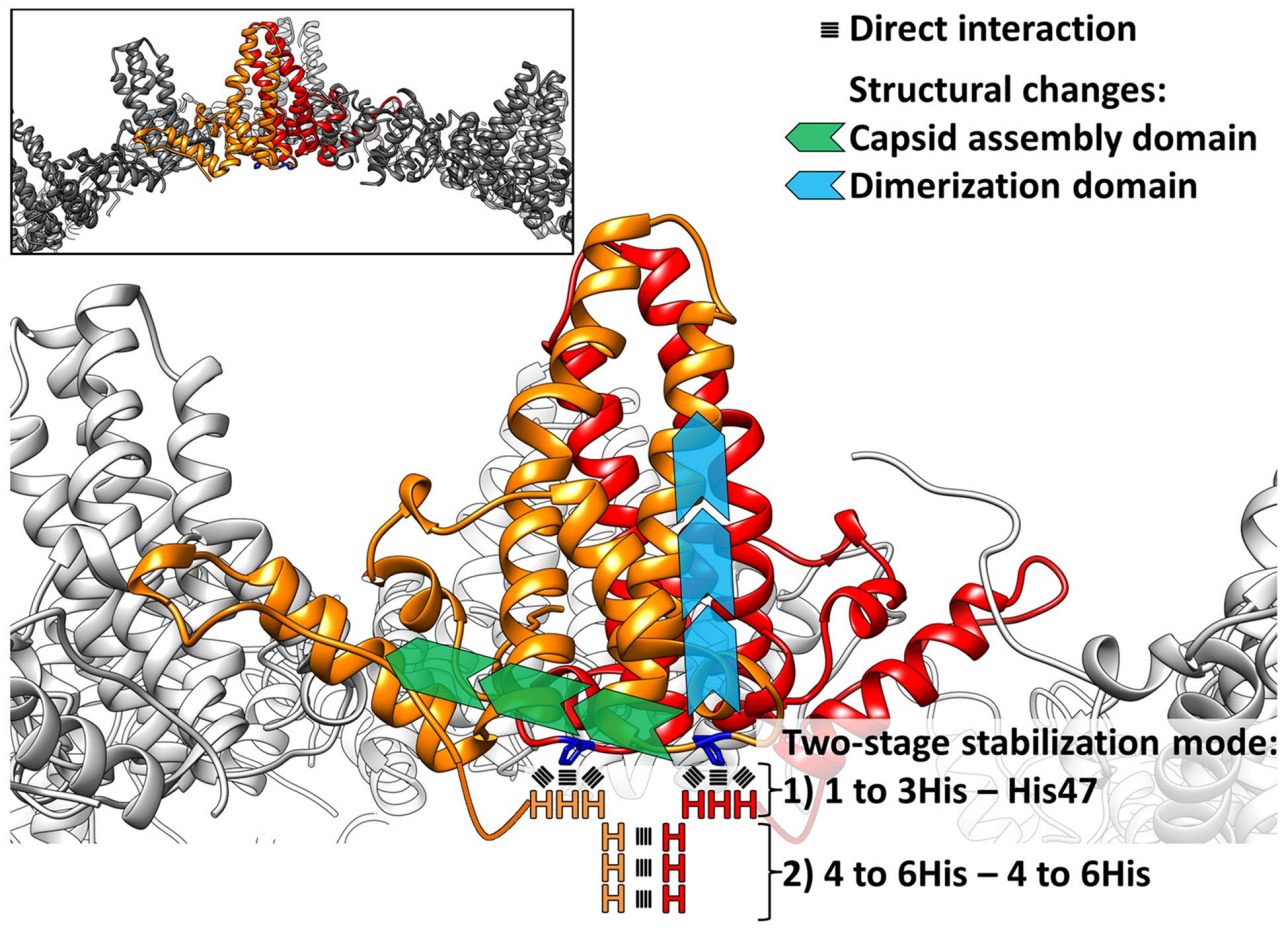
Two-stage model for the capsid stability enhancement mediated by the C-terminal polyhistidine-peptide. Schematic representation of the interactions of the C-terminal hexahistidine-peptide with the HBcAg-dimer and the proposed two-stage model for capsid stabilizing. (1) The initial three histidine moieties (HHH) of the C-terminus can interact with the conserved amino acid his47 (dark blue) of the HBcAg. This leads to structural adaptations in the capsid assembly domain (green arrows) and dimerization domain (light blue arrows) which in summary strengthen the capsid integrity. (2) The distal histidines (e.g. histidine 4–6) of two C-termini within one dimer can also interact with each other and thereby further enhance the capsid resilience. Inset show a region of the HBcAg-capsid

**Fig. 9:**
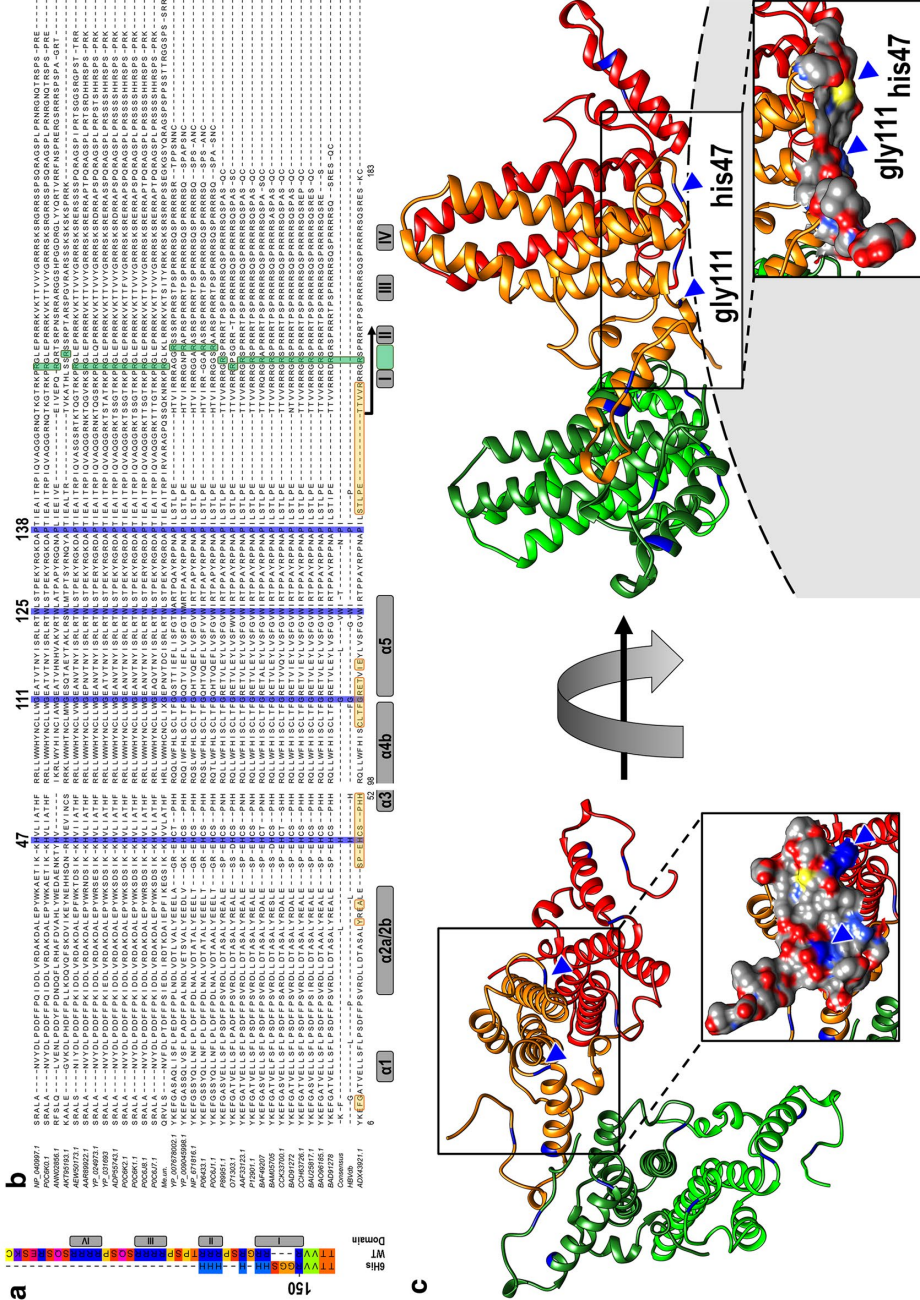
Sequence conservations and their localization in the core antigen. **a** Sequence alignment of the C-terminus of the full length HBcAg (WT) and the linker-hexahistidine-peptide (6His). Color code indicates amino acid sequence similarity according to Taylor [84]. The C-terminal domains (CTD) are marked with I–IV. **b** Multiple sequence alignment of the core antigen proteins was performed with the program “T-Coffee”, and the positions in the human core protein are indicated [9]. Sequences from hepadnaviruses of different origin and diverse tolerable human HBcAg mutation variants were used (with the following sequences: NP_040997.1 heron HBV; P0C6K0.1 heron hepatitis virus; ANN02856.1 tibetan frog hepadnavirus; AKT95193.1 white sucker HBV; AEW50173.1 parrot hepatitis B virus; AAR89922.1 ross’s goose hepatitis B virus; YP_024973.1 sheldgoose hepatitis B virus; YP_031693 snow goose HBV; ADP55743.1 duck HBV; P0C6K2.1 duck hepatitis virus white Shanghai; P0C6K1.1 duck hepatitis virus strain China; P0C6J8.1 hepatitis virus duck; P0C6J7.1 hepatitis virus 2 duck; Me.un. *Melopsittacus undulatus* BHBV; YP_007678002.1 bat HBV; YP_009045998.1 horseshoe bat hepatitis B virus; NP_671816.1 woodchuck HBV; P06433.1 woodchuck hepatitis virus 2; P0C6J1.1 ground squirrel hepatitis; P89951.1 gibbon HBV; O71303.1 woolly monkey hepatitis virus; AAF33123.1 orangutan HBV; P12901.1 chimpanzee HBV; BAF49207 HuHBV genotype H; BAM05705 HuHBV genotype G; CCK33700.1 HuHBV genotype F; BAD91272 HuHBV genotype E; CCH63726.1 HuHBV genotype D; BAU25817.1 HuHBV genotype C; BAO96185.1 HuHBV genotype B; BAD91278 HuHBV genotype A; ADX43921.1 sequence of the generated chimeric HBcAg variants). Consensus represents a consensus sequence summary of more than 200 HBcAg sequence substitutions [9]. HBVdb represents a consensus sequence extracted from 1394 human HBcAg variants [85]. Highly conserved residues are colored in blue. The localization of amino acids which have a luminal surface exposer was highlighted in yellow boxes. The HBcAg domain structure is indicated by gray boxes according to [9, 56]. Black arrows indicate the sequence segment which is covered by the C-terminal linker-hexahistidine-peptide used for the alignment in **a**. **c** Two HBcAg dimers (PDB ID: 3J2V) oriented as in the capsid were visualized. Left, a luminal view is shown, whereas the right view show a lateral perspective (in gray the lumen of the capsid is indicated). In blue, the positions of the four highly conserved residues identify in the sequence alignment in **b** are highlighted. The highly conserved residues (his47 and gly111) which localize on the inner particle surface are indicated by blue arrows. The insets show an atomic surface model of each view

The exact localization of the C-terminal linker-hexahistidine-peptide could not be determined yet. The crystal structure of the HBcAg WT (terminates at position 149) revealed that the amino acid visualized last is thr142, which resides on the basis of the dimer and inside of the VLP [13]. From this position, 17 aa of the C-terminal peptide including linker and hexahistidine-peptide (LPETTVVRGGSHHHHHH) was not yet solved at atomic resolution. We and others presented data which strongly supported the internal localization of the C-terminal polyhistidine-peptide: (I) a luminal localization is reported for C-termini of HBcAg WT VLPs which were directly related to our HBcAg-VLP [27–31, 48]. (II) Our experimental data based on cryo-electron microscopy and biolayer interferometry support this internal localization of the C-terminus also in chimeric HBcAg-VLPs. (III) MD simulations may give detailed insights into the mode of molecular interactions of a hexahistidine-peptide [49]. The combination of MD simulations with empirical data strengthens the significance of the simulations. In addition, medium resolution cryo-electron microscopy density maps, when translated into scoring functions, may be combined with physical or empirical potentials to guide conformational search and thus derive structural models of the C-terminus with high accuracy and confidence [50]. Our data are in agreement with a mutated full length HBcAg (1–183 aa) without epitope insertion. For those VLPs evidence exists, that an interaction between the C-termini of lateral dimers in the five-fold symmetry is possible [30, 51]. For our chimeric VLPs we didn’t find such a lateral interaction of C-termini of adjacent dimers (Fig. 7), which could be explained by the different length of our shorter C-terminus in comparison with the longer full length version (17 aa vs 41 aa). This suggests an adapted mode of VLP stabilization by the linker-hexahistidine-peptide. Based on our experimental data we propose a two-stage stabilization mode (Fig. 8). (1) Via the three initial histidine moieties of the linker-polyhistidine-peptide, the C-terminus is electrostatically anchored on residue his47 of the core antigen (Figs. 7c, d, 8). His47 is positioned on the interior surface of the VLP, with a luminal orientation (Figs. 7c, e, 8, 9c). Interestingly, his47 is localized between the HBcAg-dimerization domain (α3 helix) and the -capsid assembly domain, both domains being known to mainly influence the capsid stability [52–54]. The interaction of the hexahistidine-peptide with his47 may induce structural changes in the indicated HBcAg domains, which improve capsid stability (Fig. 8). First, by modulating the stability and geometry of the adjacent capsid assembly domain in a way that favors capsid assembly [52, 54]. Second, by influencing the neighboring dimerization interface, the capsid stability may be further adjusted [53, 54]. Even marginal structural variations in the respective domains, triggered by the his47 interaction, may affect the dimer–dimer binding strength. Also a minor enhancement of that dimer–dimer binding strength leads in summation (T = 3 with 90 dimers and T = 4 with 120 dimers) to a notable contribution on the capsid stability [54]. Therefore, the 3; 6 and 12His-VLPs are more resilient than the ΔHis-VLPs. We speculated that at least one histidine in the C-terminus is sufficient to stabilize the VLP. (2) In parallel, by anchoring the prolonged C-termini of the 6 or 12His-VLPs within one dimer, the terminal histidines of the polyhistidine-peptide could also interact with each other (Figs. 7e, 8). This interaction further increases the stability of the capsid by influencing the assembly or dimerization domain, possibly at an intermediate folded state [54]. Therefore, the 6 and 12His-VLPs are more stable compared to the 3His-VLPs. The extension of the linker-hexahistidine-peptide (6His) by additional six histidines (12His) only marginally increases the stability of the VLPs (Fig. 5a). Both VLP-symmetries (T = 3; T = 4) are stabilized by the C-terminal linker-polyhistidine-peptide, suggesting a general effect or mode of action in agreement with our simulations.

We want to deduce the relevance of the identified stabilization mode to related WT core particles. First, we analyzed the sequence similarity between the full lengths WT C-terminus and the utilized C-terminal linker-polyhistidine-peptide (Fig. 9a). The overall similarities between the two C-termini are high, especially between the initial six arginines of the full lengths WT, which includes the C-terminal domain I to II (CTD), and the histidine residues of the linker-polyhistidine-peptide. The CTDs are dispensable for capsid formation at high HBc concentrations and necessary for RNA-binding [51]. Detailed analysis of C-terminally truncated HBcAgs showed that the initial arginine residues of the CTD I at position 150–152 and at 154 (in front of a phosphorylation site) are only marginally involved in the RNA-binding process [55, 56]. Interestingly, an arg-ala substitution within the CTD I resulted in a reduced quantity of capsids [57]. Recent data demonstrated that at physiologically low HBc concentration, the presence of the CTD is a prerequisite for particle assembly [58]. This indicates an additional function of the arginines in the stabilization/formation process of full length WT capsid. Furthermore, it has been reported that these CTD I-clustered arginines are protease inaccessible [30], which may be explained by a close contact of the arginines to the VLP-interior, and as we assume mainly to his47. We speculate that in analogy to the stabilization mechanism of the C-terminal linker-polyhistidine-peptide, the full length WT C-terminus could also be possibly anchored by the arginines to his47 and thereby stabilize the VLP. We conclude that the linker-polyhistidine-peptide could mimic the designated part of the full length WT C-terminus, especially around arg154. The position of arg154 is not absolutely conserved, but in the range of 4 amino acids in all analyzed core sequences an arginine is detected (Fig. 9b; green boxes). This could reflect a dynamic property of this subdomain, which is already seen for the hexahistidine-peptide.

Second, we combined HBcAg substitution analysis with phylogenic analysis of different hepadnavirus lineages. Here, we identified his47 together with gly111; trp125 and pro138 as absolutely conserved core residues between hepadnaviridae from men to fish, highlighted in blue in Fig. 9b. This rare sequence conservation indicates highly crucial residues. The cruciality has already been shown for the three C-terminal residues (gly111 to pro138), which are essential for folding or capsid formation [54, 59, 60]. The functions of his47 have not been studied systematically. Only a few reports contain rudimentary indications for an indirect association of his47 with the assembly behavior of capsids [61, 62]. At present, the functions of his47 are elusive, since this residue is absolutely conserved, it most probably exhibits also an unnoticed key role for the core antigen structures, functions and stabilities. Therefore, we hypothesize a central, conserved role of his47 in the stabilization of the core antigen by a mechanism which involves arginine residues around the CTD I to II of the full length C-terminus.

With the possibility to separate chimeric T = 3- from T = 4-particles by semi-quantitative native agarose gel method, we were able to quantify T = 3/T = 4 symmetry ratios and discover that T = 3-capsids are less chemical stress resistant. A previous report indicates an equal stability for the smaller T = 3- and the larger T = 4-capsids of the HBcAg WT, adr subtype (1–140 aa) in SDS- and pH-tests, but no quantifications were carried out [46]. Böttcher and coauthors hypothesized that a switch towards smaller T = 3 particles after introducing foreign epitopes into the MIR by a higher stability of T = 3 chimeric HBcAg particles [14]. In contrast to that, we demonstrated that chemical stress (urea, pH and SDS) disturbs chimeric T = 3-capsids (originated from ayw subtype, 1–150 aa) stronger in comparison to chimeric T = 4-capsids, and independent from the C-terminal polyhistidine-peptide. Urea can be found to preferentially solvate apolar and aromatic residues, as well as the peptide backbone [63]. It is conceivable that urea lead to a disturbing of domains which influences the capsid integrity. One such a domain is the interdimer domain, it is known that the interdimer contacts in the capsid were mainly hydrophobic [52]. Urea can solvate such residues which are buried in the native state of the protein. Solvatization of these moieties lead to a weakening of the interdimer contacts, in consequence to a loss of the capsid integrity and dissociation into dimers. It is known that altering pH initiated structural changes and unfolding of proteins also for VLP [64, 65]. Physical stress (temperature, freeze/thaw and shaking) equally affected both chimeric particle symmetries. From these findings we deduce that physical stress exerts a “general” disassembling effect on chimeric VLPs, whereas chimeric T = 3 VLPs are specifically more sensitive to chemical stress. These differences must rely on the structural variations between chimeric T = 3- and T = 4-capsids. Variations, besides others (e.g. size, architecture of the VLPs), the overall dimer contacts are higher in T = 4-HBcAgΔ-capsids, 60 AB- plus 60 CD-dimers compared to 60 AB- plus 30 CC-dimers occupied in T = 3-capsids [15]. These differences may reflect the higher chemical “robustness” of the chimeric T = 4-capsids. Also it has to be considered that chimeric T = 4 particles may better tolerate the selected epitope-linker insertions and may therefore improve stress resistance compared to T = 3-capsids.

It is widely accepted that a N- or C-terminal hexahistidine-tag may improve thermal stability without being hazardous to the structure of native proteins [2, 32, 66–68]. Vogel et al. noticed a slightly higher yield of the hexahistidine-tagged chimeric HBcAg-VLPs compared to untagged HBcAg-VLPs [34], but no statistical significance was reached nor stress tests were performed. So far, according to literature surprising little work has been conducted on the stability of chimeric VLPs. To our knowledge, we can demonstrate for the first time, a broad stability analysis including physical and chemical stress on chimeric HBcAg-VLPs. We could show that chimeric ΔHis-VLPs are less stress stable than the chimeric VLPs with linker and polyhistidine-peptide, this may be already realized in TEM (higher heterogeneity), dot blot (lower signal intensity) and non-reducing PAGE (lower amounts of multimers detectable) of the ΔHis-VLP probes (Figs. 1, 2). Our stability data are in agreement with other stability data on HBcAgs which are not epitope modified. In these studies most often only one or two stress parameters were analyzed [40, 46, 69–71], yet we extended these data by exposing different VLPs to a broader set of stress parameters and adding information on freeze/thaw- and shaking-stability. It has been reported that cys61 and furthermore cys48 are the disulfide bond forming residues in HBcAg WT [53, 72, 73]. We performed MALDI-TOF-MS, immunoblot of tryptically digested VLPs in non-reduced and reduced PAGE (data not shown) and identified cys61 as the main disulfide-forming residue in chimeric HBcAg-VLPs. This disulfide-bridge has recently been identified to have an influence on the capsid kinetic in HBcAgΔ VLPs [53]. In the presence of reducing agents such as DTT, the T_m_ of the HBcAgΔ was reduced [39]. We confirmed these observations; moreover, in urea-, SDS- and freeze/thaw-tests, chimeric HBcAg-VLPs are destabilized in the presence of DTT, indicating an involvement of disulfide-linked oligomerization (cys61 and or cys48).

## Conclusions

Taken together VLPs are produced for various applications, and enhanced chimeric HBcAg-VLP-integrity is a constitutive factor that influences their suitability. The improvement of VLP-stability is of high interest for pharmacology. Studies by Ulrich as well as Lu noted problems which needed to be solved for the use of chimeric HBcAg-VLPs in human vaccines: one major problem was to receive intact particles that can withstand long-term storage [23, 74]. Additionally, an improved VLP integrity leads to an elongated immunoactivity and potentially triggers higher antibody titers. Enhancement of the integrity can be achieved by the extension of the C-terminus with a linker and a polyhistidine-peptide. The C-terminal localization of the peptide provides the benefit that it resides in the particle lumen and is thereby prone to have a lower immunogenicity [9, 33]. In conclusion, for improved stress resistance, chimeric HBcAg-VLPs should be stored in a non-reducing environment and in addition should possess a linker and at least three C-terminal histidines. We propose that three C-terminal histidines combine the stabilizing advantage with a minimal anti-histidine immunogenicity compared to six (6his) or 12 histidines (12his).

## Methods

### Cloning, expression and purification of chimeric HBcAg-VLPs

Chimeric HBcAg ΔHis (epitope insertion site 78 aa–81 aa, epitope 10 aa, 1164 Da, pI 3.8), 3His, 12His, ΔHis^2nd^ (73 aa–82 aa, 17 aa, 1835 Da, pI 4.1) and 6His^2nd^ coding sequences were ordered as synthetic genes (Geneart) and cloned into the pET-21a expression vector (Novagen). The HBcAg 6His construct consists of the HBcAgΔ (a C-terminally truncated HBcAg, which lacks the nucleotide binding domain and terminates at amino acid 150) which displays a 10mer-surface epitope of the highly selective tumor-associated cell lineage marker claudin-18 isoform 2 with additional flanking linkers (G4SG4), onto the MIR of HBcAg [38]. *E. coli* BL21-Gold (DE3) cells (Agilent Technologies) pre-cultured in LB-medium at 37 °C, were used for expression. After overnight cultivation, a large scale expression in TB-medium was inoculated. All media were supplemented with 100 µg/mL ampicillin. Chimeric HBcAg-VLP expression was induced at an OD_595_ = 2.0–2.5 by the addition of 1 mM IPTG for 4 h at 37 °C. VLPs were purified from *E. coli* lysates by heat treatment at 42 °C overnight, followed by precipitation of VLPs in the soluble fraction with 0.2–0.25 M ammonium sulfate (AMS) for 2 h at 4 °C and slow stirring. After centrifugation, the AMS-precipitate was resuspended in PBS-Tween-buffer (10 mM Na_2_HPO_4_, 1.8 mM KH_2_PO_4_, 137 mM NaCl, 2.7 mM KCl, pH 7.4 and 0.25% Tween20) by stirring overnight. For purification under native conditions, resuspended VLPs were loaded onto a HiLoad 26/60 Superdex 200 pg SEC-column (GE Healthcare), equilibrated with PBS. Chimeric HBcAg-VLPs were eluted in the void volume and stored until further usage at 4 °C. For purification under capsid dissociating conditions, resolubilized VLPs were disassembled with 3–3.5 M urea in PBS (20% Glycerol; pH 9.0) at 4 °C overnight. The preparations were loaded onto a HiLoad 26/60 Superdex 200 pg SEC-column equilibrated with 3–3.5 M urea in PBS-buffer and the fractionated HBcAg dimer-pool was collected. The reassembly into VLPs was initiated by overnight dialysis at RT into 50 mM Tris–HCl, 800 mM NaCl (pH 7.0) buffer. Reassembled VLPs were further purified and concentrated by precipitation with 0.5 M AMS, followed by centrifugation, resuspension in PBS-Tween-buffer and dialysis into PBS-buffer. VLPs were stored at 4 °C until further use. All samples were subsequently analyzed by Bis–Tris-NuPAGE (Invitrogen) or Tricine-SDS-PAGE [75]. The protein concentration of the VLP containing fractions was determined by OD measurement at 280 nm. VLP suspension was vitrified at 1 mg/mL using a CP3 cryo-blotter (Gatan, USA) on holey carbon grids. Samples were transferred to a 200 kV Polara electron microscope (FEI, Netherlands) and images were recorded on Kodak SO-163 film sheets. Images were digitized at 1.5 Å per pixel and particles were selected manually. The dataset was divided into two separate groups and image analysis was carried out using EMAN [76]. When no further resolution improvement was seen, both half datasets were compared for resolution determination. Subsequently the two half-datasets were combined for the final structural model. Docking of the crystal structures into the density map was performed using the UCSF Chimera fit to map command (http://www.cgl.ucsf.edu/chimera/). For all calculations, at least five different positions on the grid were documented and evaluated with ImageJ 1.45e software [77].

### Chemical and physical stress tests

All stress tests were performed with VLPs purified under native (data not shown) and dissociating conditions and showed similar results. The VLP concentrations were kept constant and assays were carried out in buffer with low ionic strength to minimize concentration and/or reassembly effects [15, 39, 52, 70]. To analyze the stabilizing effects of disulfide bridges within capsid dimers, additionally all stress tests (Figs. 3, 4) were performed in the presence of 100 mM DTT. Stress tests were carried out using purified chimeric VLPs dialyzed into Tris-buffer (50 mM; pH 7.5). 0.275 µg/µL dialyzed VLP samples were employed for all stress tests using dot blot or NAGE for analysis. For urea-tests, a fresh urea stock-solution (10 M) was prepared, the indicated urea molarities were adjusted with Tris-buffer and the VLP samples were added to reach the final protein concentration. Samples were subsequently incubated for 24 h at RT before analysis. Urea-tests were also performed in the presence of EDTA with no significant differences (data not shown). For temperature tests, heating was performed in a PCR thermocycler (VWR) for 15 min at the specified temperatures. VLP samples were stored afterwards at 4 °C and analyzed within the next 5 h. Freeze/thaw cycles were performed for the designated cycle numbers by snap-freezing in liquid nitrogen and thawing at 25 °C in a water bath. The samples for the SDS-test were pre-incubated for 15 min in SDS-Tris-buffer at the given concentrations (w/v) before analysis. Shaking stress was performed in a thermomixer (Eppendorf) at 25 °C with 1.200 rpm for the indicated time durations. The pH stability was carried out by overnight dialysis into buffers with various pH values. The pH of the buffers was monitored and did not change by more than ± 0.2. Buffers were exchanged at least three times with each 150-fold of excess volume. Dithiothreitol (DTT) was added as a reductant to the indicated assays 5 min before the tests were performed.

### Native agarose gel electrophoresis (NAGE), dot blot, immunoblot and dynamic light scattering (DLS)

Native agarose gel electrophoresis (NAGE) was performed using standardized 2.6% agarose gels poured in custom made equipment ensuring a homogeneous, smooth, plane surface and an equal thickness of the gels. 7 µg of each chimeric VLP sample were loaded per well and separated for 13 ± 1 h at 50 V in TAE buffer at 4 °C. Gel staining was done with fresh PageBlue colloidal Coomassie staining solution (Thermo Fisher Scientific) for 30 min, followed by overnight destaining with distilled water until a homogeneous background was obtained. The linearity between protein band intensity/protein quantity was calculated at R^2^ ≥ 0.98 for 6His and ΔHis-VLPs (0–8.5 µg protein). The inter-assay variance showed a maximum deviation of ± 3% (n = 3). For comparative dot blot analysis, 0.27 µg (all constructs with histidine-peptide) or 0.54 µg (ΔHis) of VLP samples were dotted onto a nitrocellulose membrane (Pall), followed by membrane blocking, washing and incubation for 1 h at RT with a HBcAg particle-specific, 1:10,000 diluted monoclonal antibody (mAb3120; Institute of Immunology, Tokyo, Japan) [42]. Bound antibodies were detected by incubation with species-specific, HRP-conjugated secondary IgG antibodies (Jackson ImmunoResearch). After repeated washes, the membranes were incubated with enhanced chemiluminescence solution (Thermo Fisher Scientific) and signals were documented with a luminescent Imager (ImageQuant LAS 4000, GE Healthcare). Immunoblotting of NuPAGE-separated samples was performed using PVDF membranes (Millipore). After separation, transfer and blocking of the membranes, incubation with monoclonal primary antibodies against the hexahistidine-peptide or HBcAg (mAb aa2–10) were carried out for 1 h at RT [78, 79]. Secondary antibody incubation and signal detection was done as described for the dot blot experiments. Dynamic light scattering (DLS) was used to characterize the different VLP samples for particle size distribution in the nm size range. DLS was measured using the Nicomp 380 DLS system (PSS Nicomp) according to standard examinations. 0.5 mg/mL VLP samples were analyzed in 200 µL volume, for 10 min at 25 °C in glass capillaries. The hydrodynamic mean-diameter of the particles was analyzed and only values with sufficient scattering intensities were plotted into the graphs.

### Densitometric analysis of Coomassie stained native agarose gels

Densitometric analysis of stained native agarose gels was done with four (6His) or three (ΔHis) independent VLP purifications and at least 4–10 stained agarose gels were quantified for each stress test. The pH-test was performed with only two independent purifications for both constructs. Semi-quantitative analysis of colloidal Coomassie stained protein band intensities was performed using a high-resolution image scanning system (Microtek ScanMaker i800) and the evaluation software ImageQuant TL version 7.0 (GE Healthcare). To ensure comparability, the complete NAGE procedure (casting of the gel, loading, separation, stain-/destaining process, scanning process) was standardized. Quantification was only done with gels showing a clear separation of T = 3-/T = 4-symmetries and a uniform background staining. Signal intensity (volume values) minus background was calculated for each protein band and normalized against untreated reference samples on the same NAGE (marked in Figs. 3, 4 with *) and the mean and standard deviation was generated.

### FortéBIO Octet analysis

The hexahistidine-peptide accessibility within assembled or denatured chimeric VLPs was analyzed using aminopropylsilane (APS) sensors on a FortéBIO Octet Red system (Pall Life Sciences). 4.5 µg/mL of assembled VLPs (6His or ΔHis) and 20 µg/mL of denatured (8 M urea) VLPs were used for loading routine (200 µL per sensor). Loading was terminated manually when all sensors reached the same response-rate. Sensors were blocked after loading with 5% BSA in PBS-buffer for 300 s. The His6-peptide antibody was used at concentrations of 5, 7.5 and 10 nM. The HBcAg particle-specific antibody mAb3120 was used at a concentration of 50 nM. Data were processed and analyzed using the Octet Data Analysis 7.0 software. Program: baseline: 1000 rpm for 180 s; load: 1000 rpm for 600–1200 s; baseline: 1000 rpm for 180 s; blocking: 1000 rpm for 300 s; association: 1000 rpm for 600 s; dissociation: 1000 rpm for 600 s.

### His-peptide protein simulations

For the simulation of the chimeric VLP C-terminus including the hexahistidine-peptide, a 7 Å density map derived from cryo-electron microscopy images of 6His-VLPs was used as described previously [38, 80]. The atomic structure (PDB ID: 1QGT) lacking the polyhistidine-peptide was fitted into the density map. To this aim, first, Monte Carlo (MC) simulations were performed using a filtered density map. Initially fully expanded, luminal peptide (LPETTVVRGGSHHHHHH) conformations were created before the addition of side chains using the psfgen tool [81]. The MC simulations were performed over peptide dihedrals. The energy function was a linear combination of the transformed density, Coulomb, van der Waals and knowledge based potentials for scoring solvation and the backbone conformation [82]. Second, a molecular dynamics (MD) simulation using the filtered density map was carried out. Starting from MC conformations, the final fit of the C-termini into the density map was performed exploiting the MDFF (Molecular Dynamics Flexible Fitting) tool in using the CHARMM force field (scaling factor: 0.15 of the density potential) and ran for one million steps NAMD [83]. Third, free MD simulations without the density map present were calculated. For each unit, two million steps of free MD with generalized born implicit solvent were performed. This procedure was done with five different clippings from the capsid, comprising the C-termini. Each clipping-unit was composed of 10 monomers, respectively five dimers forming a pore-like geometry to which we will refer as penta-dimer (Fig. 7a, b). This should provide a realistic environment for the simulation of the C-termini, resulting in a total of 25 loop models. In the simulations of the penta-dimer only the C-termini, respectively the hexahistidine-peptides facing the pore regions were extended and simulated. Molecular graphics images were produced using the UCSF-Chimera software.

## Additional files

**Additional file 1: Table S1.** Quality control of chimeric VLPs. **Table S2.** Mass spectrometric analysis (MALDI-TOF-MS) of tryptic fragments derived from PAGE-purified 6His (bold) and ΔHis (underlined) VLPs. **Figure S1.** Graphical evaluation of densitometric analysis of the monomeric protein bands. Monomer protein bands intensities of 0 mM DTT were set to zero (background) and max was set to 100 % (complete reduction) see Fig. 2b. RSI: relative signal intensity. **Figure S2.** Effects of chemical or physical stress on VLP stability analyzed by dynamic light scattering (DLS). Chimeric 6His-VLPs (6His) and ΔHis-VLPs (ΔHis) were A) chemically or B) physically stressed in the absence (black and light grey) or presence (+DTT, dark grey and white) of 100mM DTT and subsequently analyzed with DLS. The mean hydrodynamic diameters of all stressed VLP samples are analyzed and only measurements with adequate scattering intensities were plotted into the graphs. Standard deviations are indicated by error bars. **Figure S3.** Graphical representation of T=3/T=4-symmetry-ratios of different stress tests. T=3/T=3-symmetry-ratios of chemically (A) or physically (B) stress test were calculated from normalized densitometric measurements Figs. 3, 4. Controls subsume all reference VLP samples marked with * in Figs. 3, 4. Treated subsume all the different processed VLP samples. Values with >50 % SD were excluded from the calculations, except adjacent values had >50 % differences. Blue rectangles mark the range of the untreated control T=4/T=3 ratios (*) without DTT.

**Additional file 2: Movie S1.** Simulated, internal localization of the hexahistidine-peptide in 6His-VLP. Capsid density map (gray) and fitted crystal structure of a HBcAg dimer (pdb: 1QGT, ribbons). Penta-dimer structure in colored ribbons, green distal monomers and rainbow colors proximal monomers from which the C-terminal ends (red) are simulated (compare Fig. 7a, b).

## Abbreviations

AMS: ammonium sulfate
APS: aminopropylsilane
DTT: 1,4-dithiothreitol
DLS: dynamic light scattering
HBcAg-VLP: hepatitis B virus core antigen virus-like particles
NAGE: native agarose gel electrophoresis
SDS: sodium dodecyl sulfate
TEM: transmission electron microscopy
VLPs: virus-like-particles

## References

[1] Bachmann MF, Jennings GT (2010). Vaccine delivery: a matter of size, geometry, kinetics and molecular patterns. Nat Rev Immunol.

[2] Sánchez-Sánchez L, Tapia-Moreno A, Juarez-Moreno K, Patterson DP, Cadena-Nava RD, Douglas T, Vazquez-Duhalt R (2015). Design of a VLP-nanovehicle for CYP450 enzymatic activity delivery. J Nanobiotechnol.

[3] Mao C, Solis DJ, Reiss BD, Kottmann ST, Sweeney RY, Hayhurst A, Georgiou G, Iverson B, Belcher AM (2004). Virus-based toolkit for the directed synthesis of magnetic and semiconducting nanowires. Science.

[4] Lino CA, Caldeira JC, Peabody DS (2017). Display of single-chain variable fragments on bacteriophage MS2 virus-like particles. J Nanobiotechnol.

[5] Ludwig C, Wagner R (2007). Virus-like particles-universal molecular toolboxes. Curr Opin Biotechnol.

[6] Grgacic EV, Anderson DA (2006). Virus-like particles: passport to immune recognition. Methods.

[7] Chackerian B (2007). Virus-like particles: flexible platforms for vaccine development. Expert Rev Vaccines.

[8] Liu F, Ge S, Li L, Wu X, Liu Z, Wang Z (2012). Virus-like particles: potential veterinary vaccine immunogens. Res Vet Sci.

[9] Pumpens P, Grens E (2001). HBV core particles as a carrier for B cell/T cell epitopes. Intervirology.

[10] Whitacre DC, Lee BO, Milich DR (2009). Use of hepadnavirus core proteins as vaccine platforms. Expert Rev Vaccines.

[11] Böttcher B, Wynne SA, Crowther RA (1997). Determination of the fold of the core protein of hepatitis B virus by electron cryomicroscopy. Nature.

[12] Conway JF, Cheng N, Zlotnick A, Wingfield PT, Stahl SJ, Steven AC (1997). Visualization of a 4-helix bundle in the hepatitis B virus capsid by cryo-electron microscopy. Nature.

[13] Wynne SA, Crowther RA, Leslie AG (1999). The crystal structure of the human hepatitis B virus capsid. Mol Cell.

[14] Böttcher B, Vogel M, Ploss M, Nassal M (2006). High plasticity of the hepatitis B virus capsid revealed by conformational stress. J Mol Biol.

[15] Zlotnick A, Cheng N, Conway JF, Booy FP, Steven AC, Stahl SJ, Wingfield PT (1996). Dimorphism of hepatitis B virus capsids is strongly influenced by the C-terminus of the capsid protein. Biochemistry.

[16] Walker A, Skamel C, Nassal M (2011). SplitCore: an exceptionally versatile viral nanoparticle for native whole protein display regardless of 3D structure. Sci Rep.

[17] Spohn G, Bachmann MF (2008). Exploiting viral properties for the rational design of modern vaccines. Expert Rev Vaccines.

[18] Ausar SF, Foubert TR, Hudson MH, Vedvick TS, Middaugh CR (2006). Conformational stability and disassembly of Norwalk virus-like particles effect of pH and temperature. J Biol Chem.

[19] Zeltins A (2013). Construction and characterization of virus-like particles: a review. Mol Biotechnol.

[20] Borisova G, Borschukova O, Skrastina D, Dislers A, Ose V, Pumpens P, Grens E (1999). Behavior of a short preS1 epitope on the surface of hepatitis B core particles. Biol Chem.

[21] Nassal M, Skamel C, Kratz PA, Wallich R, Stehle T, Simon MM (2005). A fusion product of the complete *Borrelia burgdorferi* outer surface protein A (OspA) and the hepatitis B virus capsid protein is highly immunogenic and induces protective immunity similar to that seen with an effective lipidated OspA vaccine formula. Eur J Immunol.

[22] Ramqvist T, Andreasson K, Dalianis T (2007). Vaccination, immune and gene therapy based on virus-like particles against viral infections and cancer. Expert Opin Biol Ther.

[23] Lu Y, Chan W, Ko BY, VanLang CC, Swartz JR (2015). Assessing sequence plasticity of a virus-like nanoparticle by evolution toward a versatile scaffold for vaccines and drug delivery. Proc Natl Acad Sci USA.

[24] Billaud JN, Peterson D, Barr M, Chen A, Sallberg M, Garduno F, Goldstein P, McDowell W, Hughes J, Jones J, Milich D (2005). Combinatorial approach to hepadnavirus-like particle vaccine design. J Virol.

[25] Janssens ME, Geysen D, Broos K, De Goeyse I, Robbens J, Van Petegem F, Timmermans JP, Guisez Y (2009). Folding properties of the hepatitis B core as a carrier protein for vaccination research. Amino Acids.

[26] Walker A, Skamel C, Vorreiter J, Nassal M (2008). Internal core protein cleavage leaves the hepatitis B virus capsid intact and enhances its capacity for surface display of heterologous whole chain proteins. J Biol Chem.

[27] Zlotnick A, Cheng N, Stahl SJ, Conway JF, Steven AC, Wingfield PT (1997). Localization of the C terminus of the assembly domain of hepatitis B virus capsid protein: implications for morphogenesis and organization of encapsidated RNA. Proc Natl Acad Sci USA.

[28] Cheng N, Conway JF, Watts NR, Hainfeld JF, Joshi V, Powell RD, Stahl SJ, Wingfield PE, Steven AC (1999). Tetrairidium, a four-atom cluster, is readily visible as a density label in three-dimensional cryo-EM maps of proteins at 10–25 Å resolution. J Struct Biol.

[29] Watts NR, Conway JF, Cheng N, Stahl SJ, Belnap DM, Steven AC, Wingfield PT (2002). The morphogenic linker peptide of HBV capsid protein forms a mobile array on the interior surface. EMBO J.

[30] Selzer L, Kant R, Wang JC, Bothner B, Zlotnick A (2015). Hepatitis B virus core protein phosphorylation sites affect capsid stability and transient exposure of the C-terminal domain. J Biol Chem.

[31] Yu X, Jin L, Jih J, Shih C, Zhou ZH (2013). 3.5 Å cryoEM structure of hepatitis B virus core assembled from full-length core protein. PLoS ONE.

[32] Carson M, Johnson DH, McDonald H, Brouillette C, Delucas LJ (2007). His-tag impact on structure. Acta Crystallogr Sect D Biol Crystallogr.

[33] Zhao X, Li G, Liang S (2013). Several affinity tags commonly used in chromatographic purification. J Anal Methods Chem.

[34] Vogel M, Vorreiter J, Nassal M (2005). Quaternary structure is critical for protein display on capsid-like particles (CLPs): efficient generation of hepatitis B virus CLPs presenting monomeric but not dimeric and tetrameric fluorescent proteins. Proteins.

[35] Sahin U, Koslowski M, Dhaene K, Usener D, Brandenburg G, Seitz G, Huber C, Türeci O (2008). Claudin-18 splice variant 2 is a pan-cancer target suitable for therapeutic antibody development. Clin Cancer Res.

[36] Lyons TG, Ku GY (2017). Systemic therapy for esophagogastric cancer: targeted therapies. Chin Clin Oncol.

[37] Al-Batran S-E, Schuler MH, Zvirbule Z, Manikhas G, Lordick F, Rusyn A (2017). FAST: an international, multicenter, randomized, phase II trial of epirubicin, oxaliplatin, and capecitabine (EOX) with or without IMAB362, a first-in-class anti-CLDN18.2 antibody, as firstline therapy in patients with advanced CLDN18.2+ gastric and gastroesophageal junction (GEJ) adenocarcinoma. J Clin Oncol.

[38] Klamp T, Schumacher J, Huber G, Kühne C, Meissner U, Selmi A, Hiller T, Kreiter S, Markl J, Türeci Ö, Sahin U (2011). Highly specific auto-antibodies against claudin-18 isoform 2 induced by a chimeric HBcAg virus-like particle vaccine kill tumor cells and inhibit the growth of lung metastases. Cancer Res.

[39] Wingfield PT, Stahl SJ, Williams RW, Steven AC (1995). Hepatitis core antigen produced in *Escherichia coli*: subunit composition, conformational analysis, and in vitro capsid assembly. Biochemistry.

[40] Lee KW, Tan WS (2008). Recombinant hepatitis B virus core particles: association, dissociation and encapsidation of green fluorescent protein. J Virol Methods.

[41] Serwer P, Khan SA, Griess GA (1995). Non-denaturing gel electrophoresis of biological nanoparticles: viruses. J Chromatogr A.

[42] DiMattia MA, Watts NR, Stahl SJ, Grimes JM, Steven AC, Stuart DI, Wingfield PT (2013). Antigenic switching of hepatitis B virus by alternative dimerization of the capsid protein. Structure.

[43] Bereszczak JZ, Rose RJ, van Duijn E, Watts NR, Wingfield PT, Steven AC, Heck AJ (2013). Epitope-distal effects accompany the binding of two distinct antibodies to hepatitis B virus capsids. J Am Chem Soc.

[44] König S, Beterams G, Nassal M (1998). Mapping of homologous interaction sites in the hepatitis B virus core protein. J Virol.

[45] Kim R, Yokota H, Kim SH (2000). Electrophoresis of proteins and protein-protein complexes in a native agarose gel. Anal Biochem.

[46] Newman M, Suk FM, Cajimat M, Chua PK, Shih C (2003). Stability and morphology comparisons of self-assembled virus-like particles from wild-type and mutant human hepatitis B virus capsid proteins. J Virol.

[47] Yoon KY, Tan WS, Tey BT, Lee KW, Ho KL (2013). Native agarose gel electrophoresis and electroelution: a fast and cost-effective method to separate the small and large hepatitis B capsids. Electrophoresis.

[48] Wizemann H, von Brunn A (1999). Purification of *E. coli*-expressed HIS-tagged hepatitis B core antigen by Ni2+-chelate affinity chromatography. J Virol Methods.

[49] Lin YW, Ying TL, Liao LF (2011). Molecular modeling and dynamics simulation of a histidine-tagged cytochrome b5. J Mol Model.

[50] Lindert S, Staritzbichler R, Wötzel N, Karakaş M, Stewart PL, Meiler J (2009). EM-fold: de novo folding of alpha-helical proteins guided by intermediate-resolution electron microscopy density maps. Structure.

[51] Wang JCY, Dhason MS, Zlotnick A (2012). Structural organization of pregenomic RNA and the carboxy-terminal domain of the capsid protein of hepatitis B virus. PLoS Pathog.

[52] Ceres P, Zlotnick A (2002). Weak protein–protein interactions are sufficient to drive assembly of hepatitis B virus capsids. Biochemistry.

[53] Selzer L, Katen SP, Zlotnick A (2014). The hepatitis B virus core protein intradimer interface modulates capsid assembly and stability. Biochemistry.

[54] Alexander CG, Jürgens MC, Shepherd DA, Freund SM, Ashcroft AE, Ferguson N (2013). Thermodynamic origins of protein folding, allostery, and capsid formation in the human hepatitis B virus core protein. Proc Natl Acad Sci USA.

[55] Sominskaya I, Skrastina D, Petrovskis I, Dishlers A, Berza I, Mihailova M, Jansons J, Akopjana I, Stahovska I, Dreilina D, Ose V, Pumpens P (2013). A VLP library of C-terminally truncated hepatitis B core proteins: correlation of rna encapsidation with a Th1/Th2 switch in the immune responses of mice. PLoS ONE.

[56] Newman M, Chua PG, Tang F-M, Su P-Y, Shih C (2009). Testing an electrostatic interaction hypothesis of hepatitis B virus capsid stability by using an in vitro capsid disassembly/reassembly system. J Virol.

[57] Lewellyn EB, Loeb DD (2011). The arginine clusters of the carboxy-terminal domain of the core protein of hepatitis B virus make pleiotropic contributions to genome replication. J Virol.

[58] Ludgate L, Liu K, Luckenbaugh L, Streck N, Eng S, Voitenleitner C, Delaney WE, Hu J (2016). Cell-free hepatitis B virus capsid assembly dependent on the core protein c-terminal domain and regulated by phosphorylation. J Virol.

[59] Metzger K, Bringas RJ (1998). Proline-138 is essential for the assembly of hepatitis B virus core protein. J Gen Virol.

[60] Koschel M, Thomssen R, Bruss V (1999). Extensive mutagenesis of the hepatitis B virus core gene and mapping of mutations that allow capsid formation. J Virol.

[61] Stray SJ, Ceres P, Zlotnick A (2004). Zinc ions trigger conformational change and oligomerization of hepatitis B virus capsid protein. Biochemistry.

[62] Deres K, Schröder CH, Paessens A, Goldmann S, Hacker HJ, Weber O, Krämer T, Niewöhner U, Pleiss U, Stoltefuss J, Graef E, Koletzki D, Masantschek RN, Reimann A, Jaeger R, Gross R, Beckermann B, Schlemmer KH, Haebich D, Rübsamen-Waigmann H (2003). Inhibition of hepatitis B virus replication by drug-induced depletion of nucleocapsids. Science.

[63] Stumpe MC, Grubmüller H (2007). Interaction of urea with amino acids: implications for urea-induced protein denaturation. J Am Chem Soc.

[64] O’Brien EP, Brooks BR, Thirumalai D (2012). Effects of pH on proteins: predictions for ensemble and single-molecule pulling experiments. J Am Chem Soc.

[65] Samandoulgou I, Hammami R, Rayas RM, Fliss I, Jean J (2015). Stability of secondary and tertiary structures of virus-like particles representing noroviruses: effects of pH, ionic strength, and temperature and implications for adhesion to surfaces. Appl Environ Microbiol.

[66] de Li F, Feng L, Hou YJ, Liu W (2013). The expression, purification and crystallization of a ubiquitin-conjugating enzyme E2 from *Agrocybe aegerita* underscore the impact of His-tag location on recombinant protein properties. Acta Crystallogr Sect F Struct Biol Cryst Commun.

[67] Khan F, Legler PM, Mease RM, Duncan EH, Bergmann-Leitner ES, Angov E (2012). Histidine affinity tags affect MSP1(42) structural stability and immunodominance in mice. Biotechnol J.

[68] Taylor EJ, Goyal A, Guerreiro CI, Prates JA, Money VA, Ferry N, Morland C, Planas A, Macdonald JA, Stick RV, Gilbert HJ, Fontes CM, Davies GJ (2005). How family 26 glycoside hydrolases orchestrate catalysis on different polysaccharides: structure and activity of a *Clostridium thermocellum* lichenase, CtLic26A. J Biol Chem.

[69] Bundy BC, Franciszkowicz MJ, Swartz JR (2008). *Escherichia coli*-based cell-free synthesis of virus-like particles. Biotechnol Bioeng.

[70] Singh S, Zlotnick A (2003). Observed hysteresis of virus capsid disassembly is implicit in kinetic models of assembly. J Biol Chem.

[71] Nath N, Hickman K, Nowlan S, Shah D, Phillips J, Babler S (1992). Stability of the recombinant hepatitis B core antigen. J Clin Microbiol.

[72] Nassal M, Rieger A, Steinau O (1992). Topological analysis of the hepatitis B virus core particle by cysteine-cysteine cross-linking. J Mol Biol.

[73] Zheng J, Schödel F, Peterson DL (1992). The structure of hepadnaviral core antigens. Identification of free thiols and determination of the disulfide bonding pattern. J Biol Chem.

[74] Ulrich R, Nassal M, Meisel H, Krüger DH (1998). Core particles of hepatitis B virus as carrier for foreign epitopes. Adv Virus Res.

[75] Kapinos LE, Schumacher J, Mücke N, Machaidze G, Burkhard P, Aebi U, Strelkov SV, Herrmann H (2010). Characterization of the head-to-tail overlap complexes formed by human lamin A, B1 and B2 “half-minilamin” dimers. J Mol Biol.

[76] Tang G, Peng L, Baldwin PR, Mann DS, Jiang W, Rees I, Ludtke SJ (2007). EMAN2: an extensible image processing suite for electron microscopy. J Struct Biol.

[77] Collins TJ (2007). ImageJ for microscopy. Biotechniques.

[78] Bichko V, Schödel F, Nassal M, Gren E, Berzinsh I, Borisova G, Miska S, Peterson DL, Gren E, Pushko P, Will H (1993). Epitopes recognized by antibodies to denatured core protein of hepatitis B virus. Mol Immunol.

[79] Zentgraf H, Frey M, Schwinn S, Tessmer C, Willemann B, Samstag Y, Velhagen I (1995). Detection of histidine-tagged fusion proteins by using a high-specific mouse monoclonal anti-histidine tag antibody. Nucleic Acids Res.

[80] Gatsogiannis C, Markl J (2009). Keyhole limpet hemocyanin: 9-Å CryoEM structure and molecular model of the KLH1 didecamer reveal the interfaces and intricate topology of the 160 functional units. J Mol Biol.

[81] Phillips JC, Braun R, Wang W, Gumbart J, Tajkhorshid E, Villa E, Chipot C, Skeel RD, Kale L, Schulten K (2005). Scalable molecular dynamics with NAMD. J Comput Chem.

[82] Woetzel N, Karakaş M, Staritzbichler R, Müller R, Weiner BE, Meiler J (2012). BCL: score-knowledge based energy potentials for ranking protein models represented by idealized secondary structure elements. PLoS ONE.

[83] Trabuco LG, Villam E, Mitra K, Frank J, Schulten K (2008). Flexible fitting of atomic structures into electron microscopy maps using molecular dynamics. Structure.

[84] Taylor WR (1997). Residual colours: a proposal for aminochromography. Protein Eng.

[85] Hayer J, Jadeau F, Deléage G, Kay A, Zoulim F, Combet C (2013). HBVdb: a knowledge database for hepatitis B virus. Nucleic Acids Res.

