# Supplementary material for: Enhanced stability of a chimeric hepatitis B core antigen virus-like-particle (HBcAg-VLP) by a C-terminal linker-hexahistidine-peptide
Source: J Nanobiotechnology. 2018 Apr 13;16:39. doi: 10.1186/s12951-018-0363-0 (PMC5897928; doi:10.1186/s12951-018-0363-0)
Supplement: Supplementary file 1 — Additional file 1: Table S1. Quality control of chimeric VLPs. Table S2. Mass spectrometric analysis (MALDI-TOF-MS) of tryptic fragments derived from PAGE-purified 6His (bold) and ΔHis (underlined) VLPs. Figure S1. Graphical evaluation of densitometric analysis of the monomeric protein bands. Monomer protein bands intensities of 0 mM DTT were set to zero (background) and max was set to 100 % (complete reduction) see Fig. 2b. RSI: relative signal intensity. Figure S2. Effects of chemical or physical stress on VLP stability analyzed by dynamic light scattering (DLS). Chimeric 6His-VLPs (6His) and ΔHis-VLPs (ΔHis) were A) chemically or B) physically stressed in the absence (black and light grey) or presence (+DTT, dark grey and white) of 100mM DTT and subsequently analyzed with DLS. The mean hydrodynamic diameters of all stressed VLP samples are analyzed and only measurements with adequate scattering intensities were plotted into the graphs. Standard deviations are indicated by error bars. Figure S3. Graphical representation of T=3/T=4-symmetry-ratios of different stress tests. T=3/T=3-symmetry-ratios of chemically (A) or physically (B) stress test were calculated from normalized densitometric measurements Figs. 3, 4. Controls subsume all reference VLP samples marked with * in Figs. 3, 4. Treated subsume all the different processed VLP samples. Values with >50 % SD were excluded from the calculations, except adjacent values had >50 % differences. Blue rectangles mark the range of the untreated control T=4/T=3 ratios (*) without DTT. [file 12951_2018_363_MOESM1_ESM.pdf]

Enhanced stability of a chimeric hepatitis B core antigen virus-like-particle (HBcAg-VLP) by a C-terminal linker-hexahistidine-peptide

Jens Schumacher[1,2], Tijana Bacic[1,2], René Staritzbichler[5], Matin Daneschdar[2], Thorsten Klamp[1,2], Philipp Arnold[3¦], Sabrina Jägle[1,2], Özlem Türeci[4], Jürgen Markl[3] and Ugur Sahin[1,2,5,*]

[1]Biopharmaceutical New Technologies (BioNTech) Protein Therapeutics Corporation, An der Goldgrube 12, D-55131 Mainz, Germany

[2]Department of Internal Medicine III, Translational and Experimental Oncology, University Medical Center of Johannes Gutenberg University, Langenbeckstrasse 1, D-55131 Mainz

[3]Institute of Zoology, Johannes Gutenberg University, Johannes-von-Müller-Weg 6, D-55128 Mainz, Germany

[4]Ganymed Pharmaceuticals AG, An der Goldgrube 12, D-55131 Mainz, Germany

[5]TRON Translational Oncology, University Medical Center of Johannes Gutenberg University, TRON gGmbH, Freiligrathstrasse 12, D-55131, Mainz, Germany

¦ Present Addresses: Anatomical Institute, Otto-Hahn Platz 8, D-24118 Kiel

* To whom correspondence should be addressed: Ugur Sahin, Translational Oncology (TRON), Freiligrathstrasse 12, D-55131 Mainz, Germany; E-mail address: sahin@uni-mainz.de

ADDITIONAL FILES FOR PUBLICATION

	6His	ΔHis
Host cell DNA [pg/mL]	17	100
Purity [%]	96	93
Endotoxin [EU/mg]	16	236

Additional file 1: Table S1. Quality control of chimeric VLPs.

MDIDPYKEFGATVELLSFLPSDFFPSVRDLLDTASALYREALESPEHCSPHHTALRQAILCW
GELMTLATWVGVNLEDGGGGSGGGGTQDLYNNPVTGGGGSGGGGSR**DLVVSYVNTNMGLK**FR
QLLWFHISCLTFGR**ETVIEYLVSFGVWIRTPPAYRPPNAPILSTLPETTVVRGGSHHHHHH**

Additional file 1: Table S2. Mass spectrometric analysis (MALDI-TOF-MS) of tryptic fragments derived from PAGE-purified 6His (bold) and ΔHis (underlined) VLPs.

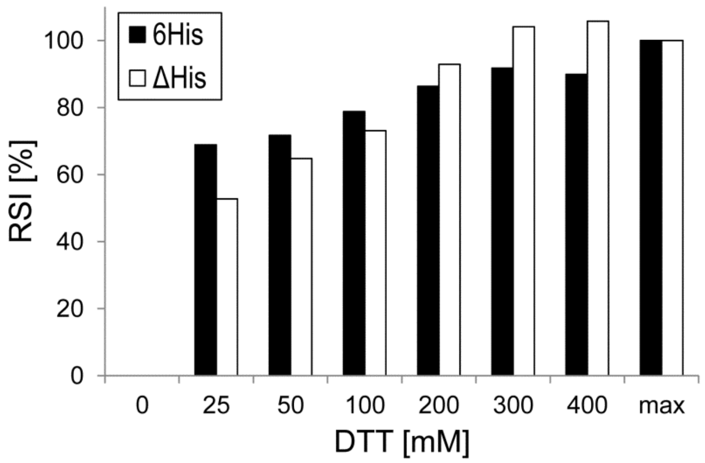

Additional file 1: Figure S1. Graphical evaluation of densitometric analysis of the monomeric protein bands. Monomer protein bands intensities of 0 mM DTT were set to zero (background) and max was set to 100% (complete reduction) see Fig. 2B. RSI: relative signal intensity.

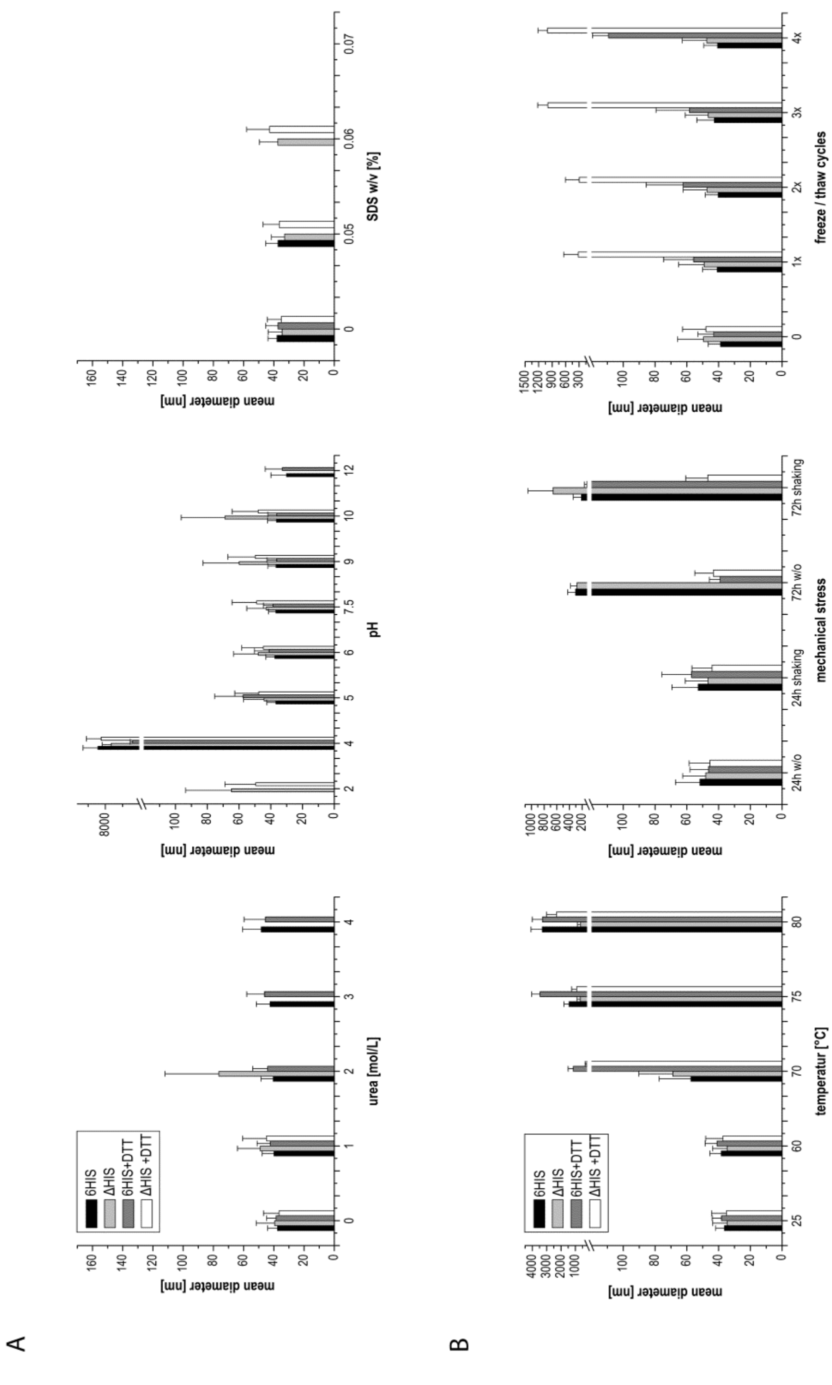

Additional file 1: Figure S2. Effects of chemical or physical stress on VLP stability analyzed by dynamic light scattering (DLS). Chimeric 6His-VLPs (6His) and ΔHis-VLPs (ΔHis) were A) chemically or B) physically stressed in the absence (black and light grey) or presence (+DTT, dark grey and white) of 100mM DTT and subsequently analyzed with DLS. The mean hydrodynamic diameters of all stressed VLP samples are analyzed and only measurements with adequate scattering intensities were plotted into the graphs. Standard deviations are indicated by error bars.

A

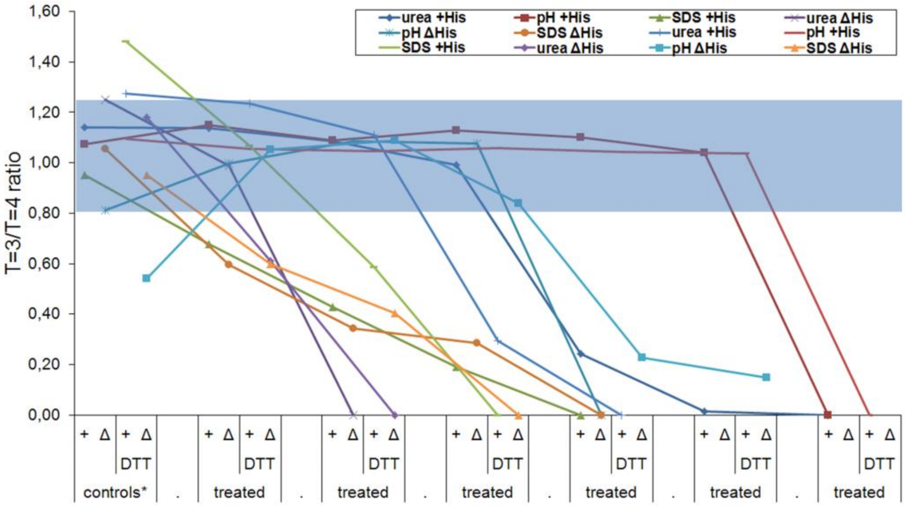

B

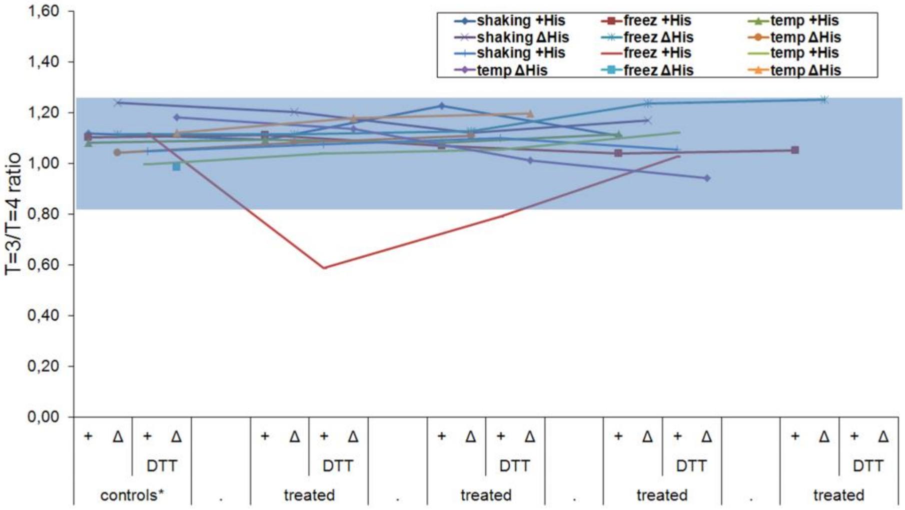

Additional file 1: Figure S3. Graphical representation of T=3/T=4-symmetry-ratios of different stress tests. T=3/T=3-symmetry-ratios of chemically (A) or physically (B) stress test were calculated from normalized densitometric measurements Fig. 3/4. Controls subsume all reference VLP samples marked with * in Fig. 3/4. Treated subsume all the different processed VLP samples. Values with >50 % SD were excluded from the calculations, except adjacent values had >50 % differences. Blue rectangles mark the range of the untreated control T=4/T=3 ratios (*) without DTT.
